# Expanded CAG/CTG repeats resist gene silencing mediated by targeted epigenome editing

**DOI:** 10.1093/hmg/ddab255

**Published:** 2021-09-07

**Authors:** Bin Yang, Alicia C Borgeaud, Marcela Buřičová, Lorène Aeschbach, Oscar Rodríguez-Lima, Gustavo A Ruiz Buendía, Cinzia Cinesi, Alysha S Taylor, Tuncay Baubec, Vincent Dion

**Affiliations:** Center for Integrative Genomics, Faculty of Biology and Medicine, University of Lausanne, Lausanne, Switzerland; Center for Integrative Genomics, Faculty of Biology and Medicine, University of Lausanne, Lausanne, Switzerland; UK Dementia Research Institute at Cardiff University, Cardiff, UK; Center for Integrative Genomics, Faculty of Biology and Medicine, University of Lausanne, Lausanne, Switzerland; Center for Integrative Genomics, Faculty of Biology and Medicine, University of Lausanne, Lausanne, Switzerland; Center for Integrative Genomics, Faculty of Biology and Medicine, University of Lausanne, Lausanne, Switzerland; Center for Integrative Genomics, Faculty of Biology and Medicine, University of Lausanne, Lausanne, Switzerland; UK Dementia Research Institute at Cardiff University, Cardiff, UK; Department of Molecular Mechanisms of Disease, University of Zurich, Zurich, Switzerland; UK Dementia Research Institute at Cardiff University, Cardiff, UK

## Abstract

Expanded CAG/CTG repeat disorders affect over 1 in 2500 individuals worldwide. Potential therapeutic avenues include gene silencing and modulation of repeat instability. However, there are major mechanistic gaps in our understanding of these processes, which prevent the rational design of an efficient treatment. To address this, we developed a novel system, ParB/ANCHOR-mediated Inducible Targeting (PInT), in which any protein can be recruited at will to a GFP reporter containing an expanded CAG/CTG repeat. Previous studies have implicated the histone deacetylase HDAC5 and the DNA methyltransferase DNMT1 as modulators of repeat instability via mechanisms that are not fully understood. Using PInT, we found no evidence that HDAC5 or DNMT1 modulate repeat instability upon targeting to the expanded repeat, suggesting that their effect is independent of local chromatin structure. Unexpectedly, we found that expanded CAG/CTG repeats reduce the effectiveness of gene silencing mediated by targeting HDAC5 and DNMT1. The repeat-length effect in gene silencing by HDAC5 was abolished by a small molecule inhibitor of HDAC3. Our results have important implications on the design of epigenome editing approaches for expanded CAG/CTG repeat disorders. PInT is a versatile synthetic system to study the effect of any sequence of interest on epigenome editing.

## Introduction

There are 14 neurological and neuromuscular phenotypes caused by the expansion of CAG/CTG repeat (1). The most common ones are myotonic dystrophy type 1 and Huntington’s disease. Their cellular phenotypes are caused by the expression of an expanded allele that generates toxic RNAs and/or peptides, which affect gene expression, splicing, and protein aggregation *in trans* (2,3). These mechanisms are thought to be worsened by somatic expansion of the expanded allele, which occurs in afflicted individuals over their lifetime (4). Indeed, longer repeats cause more severe phenotypes (5,6). Currently, there is no cure for these diseases, but modulating somatic expansion or inducing contractions are being explored as therapeutic approaches (4).

**Figure 1:**
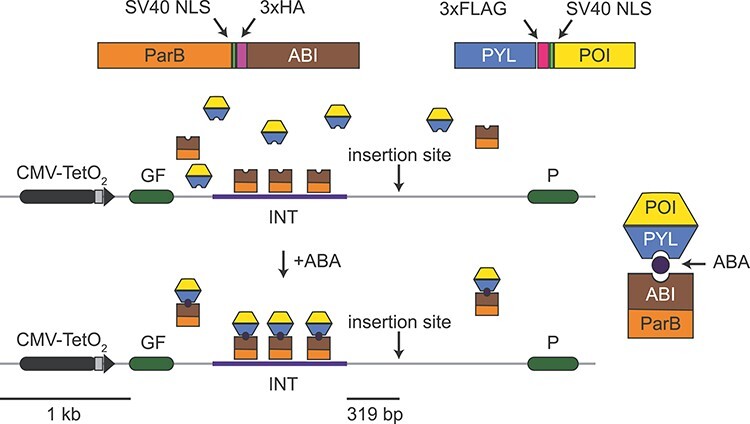
Schematic of PInT. The GFP reporter is driven by an inducible Tet-ON promoter. It contains an intron harbouring an INT sequence, which mediates the recruitment and oligomerization of ParB. We fused ParB to ABI, a plant protein that binds PYL only in the presence of abscisic acid (ABA). The PYL construct contains three tandem FLAG tags and the ParB-ABI fusion includes three tandem HA tags. They both contain SV40 nuclear localization signals. Fusing PYL to any protein of interest leads to its inducible recruitment 319 bp away from a cloning site that can be used to insert a sequence of choice. Here we chose expanded CAG/CTG repeats to test the system.

Expanded CAG/CTG repeats affect gene expression of the gene they reside in as well as neighbouring ones (7). These changes in expression are associated with gains in heterochromatin marks, including histone H3 lysine 9 methylation (H3K9me), HP1 binding and CpG methylation, as well as loss of euchromatic markers, such as CTCF binding and H3 tail acetylation (H3ac) (8–12). However, CAG/CTG repeat expansion does not appear to alter three-dimensional chromatin conformation (13). Although the heterochromatic-like state reduces the expression of the mutant allele, it does not completely abolish it (7). Furthermore, the remaining transcription through the repeat tract would be expected to support repeat instability (14). Thus, targeting the expanded allele for silencing may provide much needed symptomatic relief.

Here we asked whether epigenome editing could be harnessed to modulate gene expression and CAG/CTG repeat instability. To this end, we developed a synthetic method that enables the targeting of any peptide to a sequence of choice embedded within the intron of a fluorescent reporter. We named the system ParB/ANCHOR-mediated induced targeting (PInT). To test our system, we inserted CAG/CTG repeats within the reporter cassette such that we could monitor both their instability as well as their effect on gene expression. Using PInT, we clarified the role of two heterochromatin proteins, histone deacetylase 5 (HDAC5) and DNA methyltransferase 1 (DNMT1) in modulating repeat instability through their local recruitment. Moreover, we show, unexpectedly, that gene silencing efficiency brought about by the targeting of either HDAC5 or DNMT1 is reduced at expanded repeats compared to shorter ones. We further implicate the catalytic activity of histone deacetylase 3 (HDAC3) in helping expanded CAG/CTG repeats resist gene silencing. Our results provide novel mechanistic insights into how HDAC5 and DNMT1 impact repeat instability and uncover an unexpected effect of repeat expansion on epigenome editing.

## Results

### ParB/ANCHOR-mediated induced targeting (PInT)

We designed PInT (Fig. 1) to be modular and highly controllable. It contains a GFP mini gene that harbours two GFP exons flanking an intron of the rat *Pem1* gene (15,16). A doxycycline-inducible promoter drives the expression of the reporter. This cassette is always inserted at the same genomic location as a single copy integrant on chromosome 12 of T-Rex Flp-In HEK293 cells (13). Within the intron, we inserted a 1029 bp non-repetitive sequence, *INT*, that contains four binding sites for dimers of the *Burkholderia cenocepacia* ParB protein (17). Once bound to *INT*, ParB oligomerizes in a sequence-independent manner, recruiting up to 200 ParB molecules (18). This ParB/ANCHOR system was first used in live yeast cells to visualize double-strand break repair (17). More recently, it has been used to monitor the mobility of a genomic locus upon activation of transcription and to visualize viral replication of live mammalian cells (19–21). We made the system inducible by fusing ParB to a domain of the *A. thaliana* protein ABSCISIC ACID INSENSITIVE 1 (ABI), which dimerizes with a domain of PYRABACTIN RESISTANCE1-LIKE 1 (PYL) upon addition of abscisic acid (ABA) to the culture medium (22). ABA is a plant hormone that is not toxic to human cells, making its use especially convenient. Within 319 bp of the *INT* sequence, there is a cloning site that can be used to insert any DNA motif (Fig. 1). Fusing any protein of interest to PYL allows for full temporal control over the recruitment of a protein of interest near a DNA sequence of choice. In this case we used a CAG/CTG repeat that was either in the non-pathogenic (16 repeats) or pathogenic (≥59 repeats) range. The CAG/CTG repeats affect splicing of the reporter in a length-dependent manner, with longer repeats leading to more robust insertion of an alternative CAG exon that includes 38 nucleotides downstream of the CAG, creating a frameshift (23). Thus, we can monitor repeat size as well as changes in gene expression upon targeting any protein of choice near a CAG/CTG repeat of various sizes.

First, we determined whether the components of PInT affect the expression of the GFP reporter. We tested whether ABA changed GFP expression in GFP(CAG)_0_ cells (15). These cells carry the GFP mini gene without the *INT* sequence and no repeat in the intron (see Supplementary Material, Table S1 and Supplementary Material, Fig. S1 for details about the cell lines used and their construction). We found that treatment with up to 500 μM of ABA, which induces the dimerization between PYL and ABI (22), had no effect on GFP expression (Supplementary Material, Fig. S2A and B). We also transiently transfected GFP(CAG)_0_ cells with plasmids expressing the ParB-ABI fusion. This had no detectable effect on GFP expression (Supplementary Material, Fig. S2C). We next inserted the *INT* sequence inside the *Pem1* intron and integrated this construct using site-directed recombination, generating GFP-INT cells. These cells do not express ParB-ABI. We found that the insertion of the *INT* sequence had little, if any, discernible effect on GFP expression (Supplementary Material, Fig. S2D). We conclude that individually the components of PInT do not interfere with GFP expression.

We then stably integrated the ParB-ABI fusion into GFP-INT cells to generate GFP-INT-B cells. We found a decrease in GFP expression that correlated with higher levels of ParB-ABI (Supplementary Material, Fig. S2E–G), suggesting that the binding of ParB-ABI has a predictable effect on the expression of the GFP reporter. Because of this, we integrated ParB-ABI early in the cell line construction pipeline such that all the cell lines presented here express the same amount of ParB-ABI (Supplementary Material, Figs S1 and S3 and Supplementary Material, Table S1).

**Figure 2:**
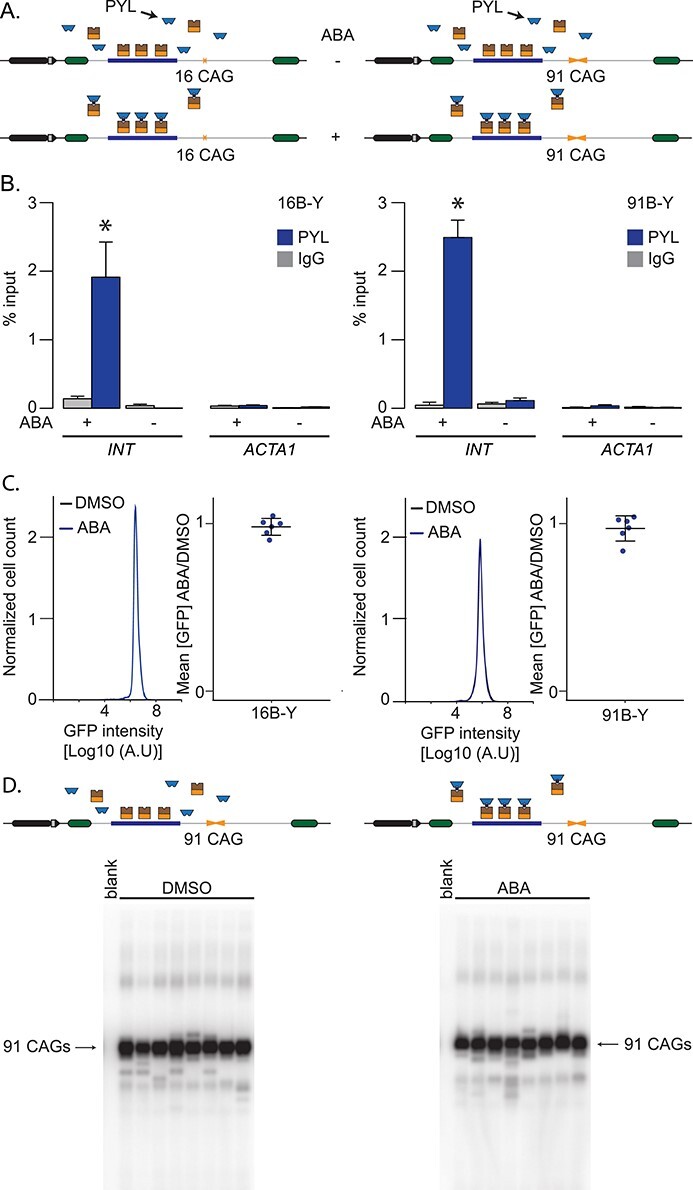
Inducible targeting of PYL at the GFP reporter. (A) Schematic representation of 16B-Y (left) and 91B-Y (right) cell lines. (B) ChIP-qPCR using antibodies against FLAG to pull down PYL at INT and ACTA1 in 16B-Y cells (left, *N* = 4) and 91B-Y cells (right, *N* = 4) after 5-day treatments with ABA or DMSO. The error bars represent the standard error. (C) Representative flow cytometry profiles after a 5-day treatment as well as quantification of the GFP expression in 16B-Y (left, *N* = 6) and 91B-Y (right, *N* = 6) cells. The error bars represent the standard deviation around the mean. (D) Representative SP-PCR blots after 30 days of continuous culture in the presence of DMSO (left) or ABA (right) in 91B-Y cells. One nanogram of DNA/reaction used in both cases.

Next, we determined the efficiency of ABA-mediated targeting PYL to the INT sequence and the consequences on GFP expression and repeat instability. We used nB-Y cells, which contain the GFP mini gene with the *INT* sequence, stably express both ParB-ABI (B) and PYL (Y), and contain *n* CAG repeats. In this case, we used either 16 CAG repeats, which is in the non-pathogenic range, or an expanded repeat of 91 triplets (Fig. 2A). Using chromatin immunoprecipitation followed by qPCR (ChIP-qPCR), we found that only 0.02% ± 0.02% and 0.1% ± 0.04% of the input *INT* DNA could be precipitated when we treated the cells with the solvent, DMSO, alone for 5 days in a cell line with 16 or 91 CAG repeats, respectively (Fig. 2B). By contrast, the addition of ABA dissolved in DMSO to the cell media increased the association of PYL to the *INT* locus significantly, reaching 1.9% ± 0.4% and 2.5% ± 0.3% of the input pulled down in 16B-Y or 91B-Y cells, respectively (Fig. 2B, *P* = 0.002 and *P* = 9 × 10^−5^, comparing DMSO and ABA, for 16B-Y and 91B-Y, respectively, using a one-way ANOVA). At the *ACTA1* locus, where there is no *INT*, the immunoprecipitated DNA remained below 0.04% regardless of the cell line or conditions used (Fig. 2B). These results demonstrate the inducible nature of the system and show that the efficiency of the targeting is similar regardless of repeat size (*P* = 0.2 comparing ABA conditions in 91B-Y and 16B-Y lines using a one-way ANOVA). Importantly, PYL targeting had no effect on GFP expression as measured by flow cytometry (Fig. 2C, *P* = 0.87 and *P* = 0.76, when comparing the mean GFP intensities upon DMSO or ABA treatment in 16B-Y and 91B-Y lines, respectively, using a one-way ANOVA). Moreover, targeting PYL to expanded CAG/CTG repeats by adding ABA to the medium of 91B-Y cells for 30 days had no effect on the frequency of repeat instability (Fig. 2D, Table 1, *P* = 0.53 comparing the number of expansions, contractions, and no change in cells treated with DMSO alone to ABA-treated cells using a χ^2^ test). ABA addition, however, decreased very slightly the magnitude of the contractions. (Supplementary Material, Fig. S4A, *P* = 0.021 using a Mann–Whitney *U* test comparing cells treated with DMSO alone or with ABA). We conclude that PInT works as an inducible targeting system and that PYL targeting is efficient and does not further affect gene expression or repeat instability.

**Table 1:** Small-pool PCR quantification after 30 days of treatment with ABA or DMSO

Cell line	Targeting^*^	Contractions	Expansions	Alleles	*P*-value − vs + ^**^
91B-Y	−	61 (9.5%)	15 (2.3%)	642	0.53
	+	53 (11.5%)	12 (2.6%)	461	
89B-Y-DNMT1	−	93 (19.2%)	49 (10.1%)	483	0.78
	+	66 (21.2%)	30 (9.7%)	310	
59B-Y-HDAC5	−	0 (0%)	14 (1.5%)	922	0.39
	+	0 (0%)	20 (2.1%)	942	

^*^: –: DMSO only, +: ABA treated.

^*^^*^: χ^2^ test with df = 2 (contractions, expansions, and stable) between − and + targeting within a cell line. For 59B-Y-HDAC5, we used a Fisher’s exact test.

### Using PInT to untangle the local versus indirect roles of chromatin modifiers

Several chromatin modifiers have been implicated in CAG/CTG repeat expansion (reviewed in (7)). These studies relied on knockout or knockdown of chromatin modifiers and could not distinguish whether factors act locally at the repeat locus (i.e. *in cis*), indirectly (i.e. *in trans*), or both. PInT is designed to evaluate these possibilities. By fusing a chromatin modifier to PYL, we can induce its local recruitment and ask whether repeat instability is affected, beyond any effect its overexpression has. The assumption is that overexpression levels are constant with and without ABA because it is done in the same cell line. If there is a difference in repeat instability between cells treated with ABA and those treated with DMSO alone, then we can conclude that the chromatin modifier acts locally. By contrast, a modifier that acts solely indirectly, for example by altering the transcriptome of a cell, will not show differences between ABA- and DMSO-treated cells. PInT is deliberately designed to compare non-targeted to targeted conditions within the same cell line as well as between lines with different repeat sizes but not between cell lines expressing different transgenes (Supplementary Material, Fig. S1).

### No evidence that DNMT1 impacts repeat instability by acting in cis

**Figure 3:**
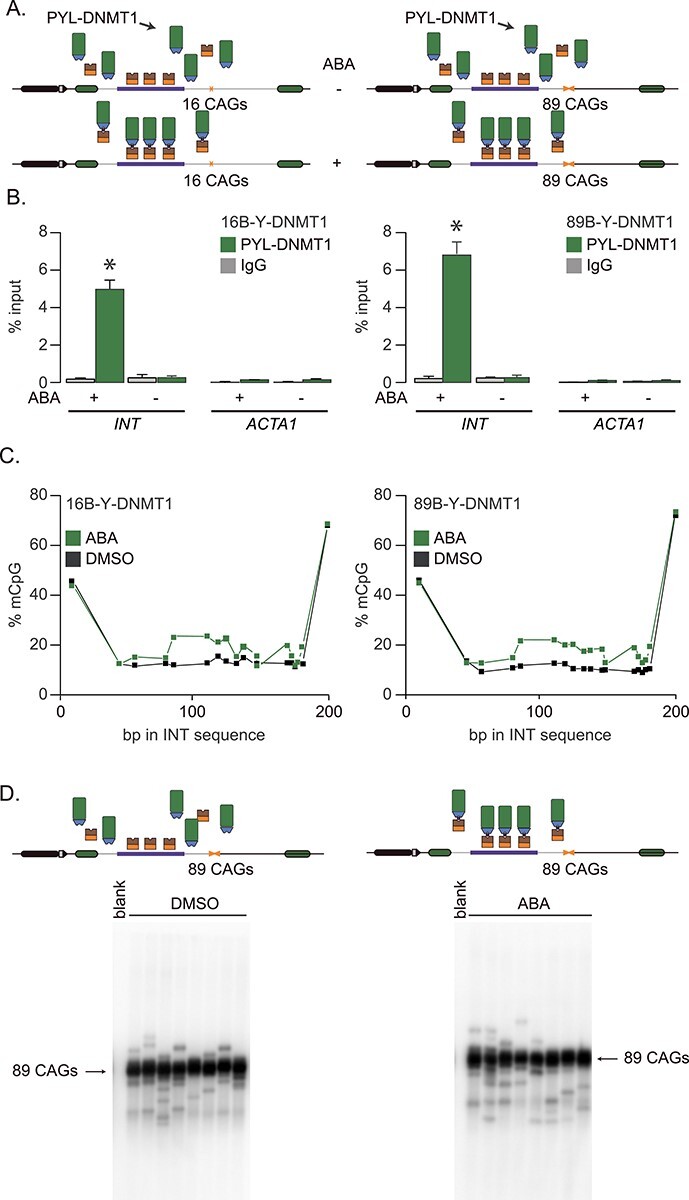
Inducible targeting of PYL-DNMT1 leads to changes in CpG methylation. (A) Schematic representation of 16B-Y-DNMT1 (left) and 89B-Y-DNMT1 (right) cell lines. (B) ChIP-qPCR using antibodies against FLAG to pull down PYL at INT and ACTA1 in 16B-Y-DNMT1 cells (left, *N* = 4) and 89B-Y-DNMT1 cells (right, *N* = 4).after 5-day treatments with ABA or DMSO. The error bars represent the standard error. (C) Bisulfite sequencing showing the percentage of methylated CpG motifs at the INT sequence in 16B-Y-DNMT1 (left) and 89B-Y-DNMT1 (right) cells in the presence of DMSO alone (black) or ABA (green) after 5-days of continuous ABA or DMSO treatment. (D) Representative SP-PCR blots after 30 days of continuous culture in the presence of DMSO (left) or ABA (right) in 89B-Y-DNMT1 cells. One nanogram of DNA/reaction used in both cases.

DNMT1 maintains DNA methylation levels during replication and repair (24). It has been implicated in preventing CAG/CTG repeat expansion in the germlines of a mouse model for spinocerebellar ataxia type 1 (25). Heterozygous *Dnmt1* mice showed lower expansion in the germlines accompanied by changes in CpG methylation flanking the repeat tract in testes and ovaries. High local CpG methylation correlated with high levels of repeat instability (25), suggesting that local levels of DNA methylation promote repeat instability. This hypothesis predicts that targeting PYL-DNMT1 will increase CpG methylation near the repeat tract and thereby increase repeat expansion frequencies. Here we tested this directly using PInT and targeted PYL-DNMT1 to 16 or 89 CAG/CTG repeats (Fig. 3A). ChIP-qPCR confirmed robust recruitment of PYL-DNMT1 to levels comparable to PYL alone (Fig. 3B). Indeed, enrichment rose upon addition of ABA from 0.3% ± 0.1% to 5.0% ± 0.5% and from 0.3% ± 0.2% to 6.8% ± 0.7% in 16B-Y-DNMT1 and 89B-Y-DNMT1 cells, respectively. The recruitment was statistically significant (*P* = 8 × 10^−5^ and *P* = 9 × 10^−5^ comparing qPCR enrichment with and without ABA in 16B-Y-DNMT1 and 89B-Y-DNMT1 lines, respectively, using a one-way ANOVA). Here again, the enrichment was not seen at the *ACTA1* locus, suggesting that it is specific to the presence of the *INT* sequence (Fig. 3B). We further determined whether targeting PYL-DNMT1 could increase levels of CpG methylation near the repeat tract. To do so, we performed bisulfite sequencing after targeting PYL-DNMT1 for 30 days. This led to changes of 10%–20% in the levels of CpG methylation, a modest increase (Fig. 3C), which is in line with the weak *de novo* methyltransferase activity of DNMT1 (for example see (26)). Similar changes in levels of CpG methylation in Dnmt1 heterozygous ovaries and testes were seen to correlate with changes in repeat instability *in vivo* (25).

Next, we assessed whether targeting PYL-DNMT1 promotes repeat expansion as predicted if the hypothesis that local CpG methylation can drive instability (25). To do so, we cultured 89B-Y-DNMT1 cells in the presence of either ABA or DMSO for 30 days, along with doxycycline to induce transcription through the repeat tract. We found no difference in the frequency of repeat instability or in the size of the changes between the ABA and DMSO conditions as measured by small-pool PCR (Table 1, Fig. 3D, Supplementary Material, Fig. S4B, *P* = 0.78 using a χ^2^ test for frequencies, *P* = 0.77 for allele size using a Mann–Whitney U test), suggesting that CpG methylation near the repeat tract is not enough to drive repeat expansion. Rather, our data argue that DNMT1 has an indirect role in CAG/CTG repeat instability.

To test whether we could detect changes in repeat size within the same time scale, we tested the effect of known modifiers of repeat instability. To this end, we cultured GFP(CAG)_101_ cells in the presence of doxycycline, which activates transcription through the repeat tract, or without it for 32 days. We saw a transcription- and time-dependent increase in expansions (Supplementary Material, Fig. S5A). Moreover, the addition of 10 μM of RGFP966, a specific HDAC3 inhibitor (27), abolished the transcription-dependent expansions and increased H3ac levels (Supplementary Material, Fig. S5A and B). These results are consistent with results obtained with other model systems (1,4,14,28–31), suggesting that our GFP reporter is reliably monitoring repeat instability. However, these experiments do not rule out that minor changes in repeat instability may be missed over that time period.

### No evidence for a local role of HDAC5 on repeat instability

HDAC3 is a class I histone acetyltransferase involved in gene silencing (33). It works together with the MutSβ complex to promote CAG/CTG repeat expansion in a human astrocyte cell line (34,35). Moreover, administration of the HDAC3 inhibitor RGFP966 to a Huntington’s disease mouse model decreased repeat expansion (31). Mechanistically, HDAC3 is thought to function in promoting repeat expansion by deacetylating MSH3, leading to its import into the nucleus where it can drive expansions (30). This model predicts that the role of HDAC3 is largely *in trans* and therefore targeting it to a repeat tract using PInT should have little effect on repeat instability. However, we could not test this hypothesis directly because we found that PYL-HDAC3 was not fully functional. Indeed, we expected that the targeting of PYL-HDAC3 would decrease GFP expression and reduce H3Ac levels. When we generated stable nB-Y-HDAC3 cells (Supplementary Material, Fig. S6A), however, we found that targeting PYL-HDAC3 in both 16B-Y-HDAC3 and 89B-Y-HDAC3 increased GFP expression by 1.5 fold (Supplementary Material, Fig. S6B, *P* = 0.002 and *P* = 0.02 using a one-way ANOVA comparing ABA and DMSO treatments in 16B-Y-HDAC3 and 89B-Y-HDAC3, respectively). The increase in GFP was accompanied by an efficient targeting of the PYL-HDAC3 fusion (Supplementary Material, Fig. S6C) and an increase in H3ac levels (Supplementary Material, Fig. S6D). We found that RGFP966 treatment did not affect the ability of PYL-HDAC3 to increase GFP expression (Supplementary Material, Fig. S6E). Moreover, transiently expressing PYL-HDAC3 in GFP-INT-B cells, without targeting, did not significantly change GFP expression (Supplementary Material, Fig. S7, *P* = 0.15 using a paired t-test comparing to controls transfected with PYL only). Together, these experiments suggest that PYL-HDAC3 does not act as expected and, thus, we could not use this line to test for a local role of HDAC3 in repeat instability.

**Figure 4:**
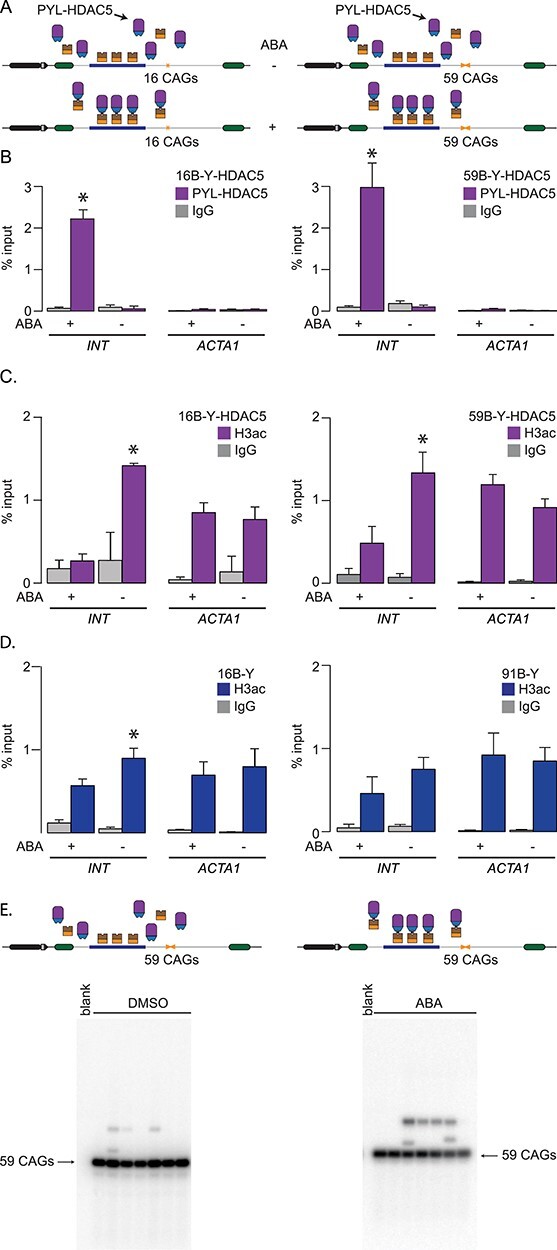
PYL-HDAC5 targeting reduces acetylation of histone H3. (A) Schematic representation of 16B-Y-HDAC5 (left) and 59B-Y-HDAC5 (right) cells. (B) ChIP-qPCR using antibodies against FLAG to pull down PYL-HDAC5 at INT and ACTA1 in 16B-Y-HDAC5 cells (left, *N* = 4) and 59B-Y-HDAC5 cells (right, *N* = 4) after a 5-day treatment with ABA or DMSO. The error bars represent the standard error. (C) ChIP-qPCR data using a pan-acetylated H3 antibody to pull down the INT and ACTA1 loci in 16B-Y-HDAC5 (left, *N* = 4) and 59B-Y-HDAC5 (right, *N* = 4) cells after a 5-day treatment with (A). The error bars represent the standard error. (D) ChIP-qPCR data using a pan-acetylated H3 antibody to pull down the INT and ACTA1 loci in 16B-Y (left, *N* = 4) and 91B-Y (right, *N* = 4) cells. The error bars represent the standard error. (E) Representative SP-PCR blots after 30 days of continuous culture in the presence of DMSO (left) or ABA (right) in 59B-Y-HDAC5 cells. One nanogram of DNA/reaction used in both cases.

Since HDAC3 works together with HDAC5 in repeat instability (32), we tested whether PYL-HDAC5 may be functional and allow us to test the hypothesis that local recruitment of PYL-HDAC5 impacts repeat instability. HDAC5 is a class IIa deacetylase associated with gene silencing and heterochromatin maintenance (33) as well as cell proliferation (34,35). It is thought to mediate histone deacetylation by recruiting other HDACs, including HDAC3 (36). We created isogenic nB-Y-HDAC5 cells that stably express a PYL-HDAC5 fusion and contain 16 or 59 CAG repeats within the GFP reporter (Fig. 4A). We found that adding ABA to the culture medium led to an increase in pull-down efficiency of PYL-HDAC5 at the *INT* locus from 0.06% ± 0.03% to 2.2% ± 0.2% in 16B-Y-HDAC5 cells and from 0.1% ± 0.1% to 3.0% ± 0.6% in 59B-Y-HDAC5 (Fig. 4B). PYL-HDAC5 targeting reduced the levels of acetylated histone H3 (H3Ac), as measured by ChIP-qPCR (Fig. 4C, *P* = 1.7 × 10^−6^ and *P* = 0.039 comparing DMSO- and ABA-treated 16B-Y-HDAC5 and 59B-Y-HDAC5, respectively, using an one-way ANOVA), consistent with a functional recruitment of PYL-HDAC5 to the *INT* sequence. This was confirmed by transiently transfecting PYL-HDAC5 in GFP-INT cells, which led to slightly lower GFP expression than those expressing PYL alone (Supplementary Material, Fig. S4A). Notably, PYL recruitment led to a marginally significant decrease in H3Ac level in 16B-Y cells but the decrease was not statistically significant in 91B-Y cells (Fig. 4D, *P* = 0.04 and *P* = 0.28 comparing the DMSO and ABA treatments in 16B-Y and 91B-Y cells, respectively, using an one-way ANOVA). Moreover, we found no significant change in acetylation upon ABA treatment at the *ACTA1* locus in either cell lines (*P* > 0.09 using a one-way ANOVA comparing H3ac levels in DMSO and ABA-treated cells). Interestingly, the H3ac levels at the *INT* sequence were similar between 16B-Y and 91B-Y (Fig. 4D, *P* = 0.44 comparing DMSO-treated 16B-Y and 91B-Y cells using a one-way ANOVA), suggesting that the H3ac levels are unaffected by the expansion. Our results show that targeting PYL-HDAC5 reduces the levels of acetylated H3 near the repeat tract.

To monitor the local effect of PYL-HDAC5 targeting on CAG/CTG repeat instability, we cultured 59B-Y-HDAC5 cells with ABA or DMSO for 30 days. We found no difference in allele-size distribution between these two treatments (Fig. 4E, Supplementary Material, Fig. S4, Table 1, *P* = 0.39 using a Fisher’s exact test, comparing ABA and DMSO-treated cells for frequencies of expansions, and *P* = 0.70 using a Mann–Whitney *U* test for changes in allele size). Therefore, we find no evidence to support the hypothesis that HDAC5 promotes repeat expansion via local changes in protein acetylation around the repeat tract.

### Gene silencing efficiency depends on CAG/CTG repeat length

We originally designed PInT to determine whether factors work *in cis* for repeat instability, yet our construct also includes a GFP reporter that can be used for monitoring gene expression. This is useful to look for chromatin modifiers that can silence expanded repeats. Indeed, finding factors that, upon targeting, can silence a gene specifically when it bears an expanded allele would open doors to novel therapeutic avenues.

**Figure 5:**
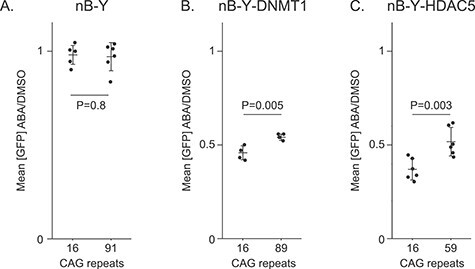
Effect of expanded CAG/CTG repeats on targeted epigenome editing. Mean GFP intensity ratios between ABA and DMSO alone after 5 days of treatment plotted for (A) 16B-Y cells (same data as in Fig. 2C, *N* = 6), 91B-Y cells (same data as in Fig. 2C, *N* = 6), (B) 16B-Y-DNMT1 (*N* = 4), 89B-Y-DNMT1 (*N* = 4), (C) 16B-Y-HDAC5 (*N* = 6), and 59B-Y-HDAC5 (*N* = 6). *P*-values were generated using one-way ANOVA.

We evaluated whether DNMT1 or HDAC5 targeting could silence a reporter bearing CAG/CTG repeats using PInT to measure GFP expression upon ABA addition (Fig. 5A). In 16B-Y-DNMT1 cells, ABA treatment decreased GFP expression by 2.2-fold compared to DMSO treatment alone. Surprisingly, ABA-induced silencing was 1.8-fold compared to DMSO alone, or 16% less efficient in 89B-Y-DNMT1 than in 16B-Y-DNMT1 cells. Although relatively small, the decrease between the two lines was statistically significant (Fig. 5B, *P* = 0.005 using a one-way ANOVA comparing the ratio of the mean GFP expression between ABA and DMSO-treated cells between the two cell lines). This was not due to PYL-DNMT1 being targeted more efficiently upon ABA addition or leading to higher levels of CpG methylation around the repeat tract in 16B-Y-DNMT1 cells compared to 89B-Y-DNMT1 cells (Fig. 3B and C). These results rather suggest that the presence of an expansion reduces the efficiency of PYL-DNMT1 to silence the reporter.

We next addressed whether this effect was specific to DNMT1. We added ABA to the medium of 16B-Y-HDAC5 cells for five days and found a reduction of GFP expression of 2.7-fold (Fig. 5A). This decrease in expression was significantly smaller in the context of an expanded repeat (Fig. 5C, Supplementary Material, Fig. S8, *P* = 0.003 comparing the decrease in expression upon ABA addition between the 16B-Y-HDAC5 and 59B-Y-HDAC5 using a one-way ANOVA). Some more mundane explanations were ruled out, including a difference in targeting efficiency of PYL-HDAC5 or changes in H3Ac levels between the cell lines (Fig. 4B and C). We also tested whether the allele length-specific effect on GFP expression required the presence of the *INT* sequence. Thus, we transiently expressed PYL-HDAC5 in GFP(CAG)_0_B cells, which have no *INT* in their GFP reporter but express ParB-ABI. Adding ABA to these cells did not affect GFP expression (Supplementary Material, Fig. S7), suggesting that the presence of the *INT* sequence is essential. Taken together, our results suggest that expanded CAG repeats resist gene silencing mediated by both DNMT1 and HDAC5.

### The N-terminal domain of HDAC5 mediates silencing

**Figure 6:**
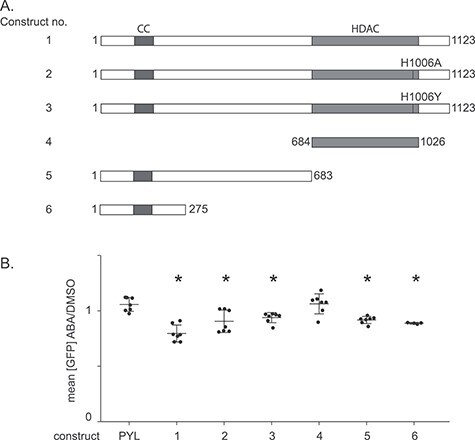
HDAC5 mediates silencing through its N-terminal. (A) Mutants and truncations of HDAC5 fused to PYL. The coiled-coil (CC) domain is indicated in purple, the deacetylase domain (HDAC) in orange. (B) ABA and DMSO treatments of 40B cells transiently transfected with plasmids containing the constructs shown in B. Construct 1: *N* = 7, *P* = 0.00001 versus PYL; construct 2: *N* = 7, *P* = 0.006 versus PYL; construct 3: *N* = 7, *P* = 0.001 versus PYL; construct 4: *N* = 7, *P* = 0.88 versus PYL; construct 5: *N* = 7, *P* = 0.0002 versus PYL; construct 6: *N* = 4, *P* = 0.0003 vs PYL. ^*^: *P* ≤ 0.01 compared to PYL targeting. The error bars show the standard deviation around the mean. *P*-values were generated using one-way ANOVA.

PInT can also be used to delineate the mechanism of gene silencing upon targeting of a chromatin modifier. To exemplify this, we sought to clarify how HDAC5 silences the reporter. Class I HDACs, like HDAC3, derive their catalytic activity *in vitro* from a conserved tyrosine residue that helps coordinate a zinc ion essential for catalysis (35). By contrast, Class IIa enzymes, like HDAC5, have a histidine instead of tyrosine at the analogous site, which considerably lowers HDAC activity (35). In fact, restoring the tyrosine at position 1006 of HDAC5 increases HDAC activity by over 30-fold (35). We reasoned that if the HDAC activity was responsible for the silencing activity, the H1006Y gain-of-function mutant should lead to a more robust silencing. Moreover, the H1006A mutant, in which the HDAC activity is dramatically reduced, would not be expected to silence the reporter. To test these predictions, we transiently transfected PYL-HDAC5 wild-type as well as H1006Y and H1006A mutants in 40B cells, which contain the GFP-INT reporter with 40 CAGs and express ParB-ABI (Fig. 6A). Overall, the effect on silencing seen upon targeting of the wild-type PYL-HDAC5 fusion was smaller when delivered by transient transfection compared to the stable cell lines. Nevertheless, the wild-type PYL-HDAC5 significantly reduced GFP expression compared to PYL alone (*P* = 0.00001 using a one-way ANOVA). In the same conditions, targeting PYL-HDAC5-H1006A or PYL-HDAC5-H1006Y both silenced the transgene compared to targeting PYL alone (Fig 6B; *P* = 0.006 and 0.002, respectively, using a one-way ANOVA), suggesting that tampering with the catalytic activity of HDAC5 does not influence silencing of our GFP reporter. Moreover, targeting PYL fused to the catalytic domain of HDAC5 did not shift GFP expression compared to PYL alone (Fig. 6B, *P* = 0.88 using a one-way ANOVA). Rather, we find that the silencing activity was contained within the N-terminal part of HDAC5, which characterizes Class IIa enzymes. Further truncations (Fig. 6A) are consistent with a model whereby the coiled-coil domain in the N-terminal part of HDAC5, which is necessary for homo- and heterodimerization of Class IIa enzymes *in vitro* (37), contains the silencing activity (Fig. 6B). It may therefore be that this domain recruits endogenous HDACs to the locus and mediate gene silencing.

### HDAC3 activity is required for the repeat size-specificity upon HDAC5-mediated silencing

**Figure 7:**
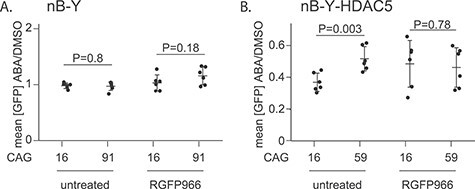
Allele-size specificity of HDAC5-mediated gene silencing requires HDAC3 activity. Quantification of GFP intensity upon targeting in the presence of 10 μM of RGFP966 or untreated (A) 16B-Y (*N* = 6 for each condition) and 91B-Y cells (*N* = 6 for each condition) and (B) 16B-Y-HDAC5 (*N* = 6 for each condition) and 59B-Y-HDAC5 cells (*N* = 6 for each condition). Note that the data for the untreated cells are the same as in Figure 5. *P*-values were generated using one-way ANOVA.

Next, we asked whether PInT could be used to gain insights into the mechanism of targeted epigenome editing. To do so, we sought to find enzymatic activities that can modify allele size-specific silencing brought about by PYL-HDAC5 targeting. To determine whether the catalytic role of HDAC3 was essential in HDAC5-mediated silencing, we repeated our experiments in nB-Y and nB-Y-HDAC5 lines in the presence of the HDAC3 inhibitor RGFP966 (Fig. 7). We found that RGFP966 had no effect on GFP expression upon PYL targeting (Fig. 7A) and did not substantially reduce the ability of PYL-HDAC5 to silence the reporter (Fig. 7B). However, it abolished the allele-length specificity of PYL-HDAC5 targeting, leading to a silencing efficiency of 2.4- and 2.5-fold in 16B-Y-HDAC5 and 59B-Y-HDAC5, respectively (Fig. 7B, *P* = 0.78 using a one-way ANOVA). This contrasts with the RGFP966-free conditions where targeting PYL-HDAC5 more effectively silenced the non-pathogenic-sized allele (Fig. 7B). We also used a two-way ANOVA to test for a significant interaction between repeat size and RGFP966 treatment. We found one for PYL-HDAC5 but not for PYL alone (*P* = 0.019 and *P* = 0.20, respectively). These results suggest expanded CAG/CTG repeats impede PYL-HDAC5-mediated silencing via the catalytic activity of HDAC3.

## Discussion

Chromatin structure impinges on every DNA-based transaction, from replication and DNA repair to transcription. Consequently, epigenome editing is being harnessed to understand basic molecular mechanisms of pathogenesis and for the development of novel therapeutic approaches (38). Epigenome editing is now most commonly carried out by fusing a chromatin modifying peptide to a catalytically dead Cas9 (dCas9). These dCas9-based approaches are highly versatile and have been used successfully to modify disease phenotypes in cells and *in vivo* (39,40). PInT is meant to complement dCas9-based approaches. Specifically, PInT offers two advantages that we have exploited here. First, it can concentrate a large number of molecules at a target site (18), independently of chromatin context (17). It is less practical with dCas9 to recruit equivalent numbers of molecules at a specific locus as it would require the use of multimerization domains, in addition to multiple sgRNAs. Second, targeting a chromatin modifying peptide to different loci can have very different effects (41,42). This highlights that DNA context affects epigenome editing in ways that are not currently understood. Here we designed PInT to isolate expanded repeats tracts from their endogenous location and concentrate on the effect of chromatin modifiers. Nonetheless, with PInT it may be possible to include *cis* elements next to the repeat tract and evaluate their effects on instability or gene expression. In fact, PInT may be used to clone any sequence of interest near the targeting site and can be utilized for a wide array of applications, beyond the study of expanded CAG/CTG repeats.

Several studies have suggested that the ectopic insertion of an expanded CAG/CTG repeat in mice could induce changes in chromatin structure in the abutting sequences. An early example was the random insertion of arrays of transgenes, each carrying 192 CAGs, which led to the silencing of the transgenes independently of the site of genomic integration (12). In addition, inserting a 40 kb human genomic region containing the *DMPK* gene along with an expansion of 600 CTGs (11), or a 13.5 Kb region containing the human SCA7 gene with 92 CAGS (10) all led to changes in chromatin marks near the expansion. It has been unclear, however, whether the presence of endogenous sequence elements, like CpG islands (43) and CTCF-binding sites (9,44), is necessary for this effect. Our data show that 91 CAGs, without the flanking sequences normally present at the *DMPK* gene from whence this repeat was cloned, does not lead to significant changes in the levels of H3ac in its vicinity. These data suggest that the flanking sequence elements may play important roles in the induction and/or maintenance of heterochromatic marks surrounding expanded CAG/CTG repeats.

PInT can be used to design peptides with enough activity to be useful in downstream epigenome editing applications. For instance, here we dissected the mechanisms of action of HDAC5 in silencing using mutants and truncations. We could quickly screen for domains and mutants that are effective in modulating gene expression. This is especially desirable in designing epigenome editing approaches with dCas9 fusions *in vivo*. A current limitation of the *S. pyogenes* Cas9 for *in vivo* applications is its large size, which is at the limit of what adeno-associated viral vectors can accommodate (45). Even with the smaller orthologues, packaging a dCas9 fusion inside a gene delivery vector is a challenge, let alone encoding the sgRNA in the same vector. Therefore, being able to trim a chromatin modifier down to its smallest active peptide may help in optimizing downstream applications and translation.

In this study, we addressed a central question for both HDAC5 and DNMT1 and their involvement in CAG/CTG repeat instability. It has been unclear what the exact roles of these two enzymes might be in repeat instability. Specifically, whether they work by modifying the local chromatin structure or they act *in trans* has remained an outstanding issue. For DNMT1, it was speculated that increases in CpG methylation surrounding the repeat tract might facilitate repeat expansion (25). The data presented here do not support such a model and rather point to an indirect role for DNMT1 in repeat instability, perhaps through changes in the transcriptome. For example, DNMT1 controls the expression of MLH1 (46), which has been shown to be important for repeat instability (47–51). It also remains possible that DNMT1 targeting did not lead to large enough changes in CpG methylation to affect repeat instability. The case of HDAC5 is possibly more complex as its partner, HDAC3, has been shown to play a role in the deacetylation of MSH3 (30), a known modifier of repeat instability (14,47,50,52–54). Although we cannot rule out that the lack of an effect *in cis* is due to the relatively low frequency of instability in the 59B-Y-HDAC5 line, the results obtained with PInT are concordant with a role for HDAC5 *in trans*. HDAC5 may help control the deacetylation of MSH3 before it binds to the repeat tract. Together, these results highlight the usefulness of PInT in understanding the mechanism of repeat instability.

The observation that expanded CAG/CTG repeats resist gene silencing is intriguing. This effect appears to be independent of which silencer is targeted as we have tried two, DNMT1 and HDAC5, which have different modes of action. We have identified an HDAC3 inhibitor, RGFP966, that abolishes the difference in repeat size upon HDAC5 targeting without affecting the silencing activity. Importantly, we cannot currently rule out that RGFP966 may inhibit other HDACs that would be responsible for this effect.

Epigenome editing, through targeting of PYL-HDAC5 or PYL-DNMT1, remained unaffected, with similar levels of deacetylation and DNA methylation levels regardless of repeat size. These results suggest that neither H3ac nor DNA methylation are good proxies for gene silencing. There are several steps towards gene silencing that could be differentially affected by the presence of a CAG/CTG repeat expansion. First, transcription is known to be impeded, at least *in vitro*, by the presence of a repeat tract (55). This is counter to the effect on gene silencing that we observed here. Alternatively, splicing may be differentially regulated by both the expanded repeats and the targeted epigenome editing. Indeed, histone marks correlate with changes in splicing patterns (56) and expanded CAG/CTG repeats are known to affect splicing (23,57). Moreover, both HDAC3 and HDAC5 interact with splicing factors (58). It is also possible that mRNA or GFP stability may contribute to the repeat-size-specific effect seen here, but the mechanism would have to be more convoluted. Ultimately, finding the HDAC3 target that mediates this effect will help understanding the mechanism of allele-specific gene silencing that we uncovered here.

The observations that expanded CAG/CTG repeats reduces the efficiency of gene silencing has implications in the design of epigenome editing approaches for expanded repeat disorders. We speculate that PInT may be adapted to screen for allele length-specific silencers, which may help design novel therapeutic options for expanded CAG/CTG repeat disorders.

## Materials and Methods

### Cell culture conditions and cell line construction

Most of the cell lines used, including all the parental lines, were genotyped by Microsynth, AG (Switzerland) and all confirmed to be HEK293.2sus. They were free of mycoplasma as assayed by the Mycoplasma check service of GATC Biotech. The cells were maintained at 37°C with 5% CO_2_ in DMEM containing 10% FBS, penicillin and streptomycin, as well as the appropriate selection markers at the following concentrations: 15 μg ml^−1^ blasticidin, 1 μg ml^−1^ puromycin, 150 μg ml^−1^ hygromycin, 400 μg ml^−1^ G418 and/or 400 μg ml^−1^ zeocin. Whereas FBS was used to maintain the cells, dialyzed calf serum was used at the same concentration for all the experiments presented here. The ABA concentration used was 500 μM, unless otherwise indicated. Doxycycline (dox) was used at a concentration of 2 μg ml^−1^ in all experiments. RGFP966 was used at a concentration of 10 μM. Notably, it is not possible to obtain several stable lines with the exact same repeat size as they are, by their nature, highly unstable. This is why we have lines with different repeat sizes. Furthermore, the sizes can change over time and upon thawing from the freezer.

A schematic of cell line construction and pedigree is found in Supplementary Material, Fig. S1, and the lines are listed in Supplementary Material, Table S1. This table includes the plasmids made for cell line construction. The levels of the transgenes are found in Supplementary Material, Fig. S3. The plasmids used for transient transfections are found in Supplementary Material, Table S2. For each cell line, single clones were isolated and tested for expression of ParB-ABI and PYL-fusions by western blotting using the protocol described before (16). Briefly, whole cell extracts were obtained, and their protein content was quantified using the Pierce BCA Protein Assay Kit (ThermoScientific). Proteins were then run onto Tris-glycine 10% SDS PAGE gels before being transferred onto nitrocellulose membrane (Axonlab). The membranes were blocked using the Blocking Buffer for Fluorescent Western Blotting (Rockland), and primary antibodies were added overnight. Membranes were then washed followed by the addition of the secondary antibody (diluted 1–2000). The fluorescent signal was detected using an Odyssey Imaging System (Li-CoR). All antibodies used are found in Supplementary Material, Table S3. Unaltered western blot images are found in Supplementary Material, Fig. S9. To assess repeat sizes, we amplified the repeat tracts using oVIN-0459 and oVIN-0460 with the UNG and dUTP-containing PCR as described (59) and then Sanger-sequenced by Microsynth AG (Switzerland). The sequences of all the primers used in this study are found in Supplementary Material, Table S4.

The ParB-INT sequence system used here is the c2 version described previously (17), except that the ParB protein was codon-optimized for expression in human cells. It is also called ANCHOR1 and is distributed by NeoVirTech. ParB-ABI (pBY-008), PYL (pAB-NEO-PYL), PYL-HDAC5 (pAB(EXPR-PYL-HDAC5-NEO)) and PYL-HDAC3 (pAB(EXPR-PYL-HDAC3-NEO)) constructs were randomly inserted and single clones were then isolated (Supplementary Material, Table S1). GFP-reporter cassettes were inserted using Flp-mediated recombination according to the manufacturer’s instruction (Thermo Scientific). Single colonies were picked and screened for zeocin sensitivity to ensure that the insertion site was correct. The cell lines, and plasmids generated and analysed in the current study are available from the corresponding author. Note that to obtain some of the plasmids, researchers will also need the permission of NeoVirTech, which owns the rights to the ANCHOR technology.

### Targeting assays

Detailed protocols of the assay and culture conditions can be found in (60). For targeting assays involving transient transfections, cells were plated onto poly-D-lysine-coated 12-well plates at a density of 6 × 10^5^ cells per well and transfected using 1 μg of DNA per well and Lipofectamine 2000 or Lipofectamine 3000 (Thermofisher Scientific). Six hours after transfection, the medium was replaced with one containing dox and ABA or DMSO. Forty-eight hours after the transfection, the cells were split, and fresh medium with dox and ABA or DMSO was replenished. On the fifth day, samples were detached from the plate with PBS + 1 mM EDTA for flow cytometry analysis.

In the case of the stable cell lines, cells were seeded at a density of 4 × 10^5^ per well in 12-well plates. The media included dox and ABA or DMSO. The medium was changed 48 h later and left to grow for another 48 h. The cells were then resuspended in 500 μl PBS + 1 mM EDTA for flow cytometry analysis.

### Flow cytometry

We used an Accuri C6 flow cytometer from BD and measured the fluorescence in at least 12 500 cells for each treatment. The raw data were exported as FCS files and analysed using FlowJo version 10.0.8r1. A full protocol is available here (60).

### Chromatin immunoprecipitation

For chromatin immunoprecipitation, the cells were treated as for the targeting experiments except that we used 10 cm dishes and 4 × 10^6^ cells. After 96 h of incubation, paraformaldehyde was added to the medium to a final concentration of 1% and the cells were incubated for 10 min at room temperature. The samples were then quenched with 0.125 M PBS-glycine for 5 min at room temperature. Samples were then centrifuged, the supernatant was discarded, and the cell pellets were washed with ice-cold PBS twice. The samples were split into 10^7^ cell aliquots and either used immediately or stored at −75°C for later use. Sonication was done using a Bioruptor for 25–30 min. DNA shearing was visualized by agarose gel electrophoresis after crosslink reversal and RNase treatment. Twenty percent of sonicated supernatant was used per IP, with 3 μg anti-FLAG (M2, Sigma), anti–PAN-acetylated H3 (Merck), or anti-IgG (3E8, Santa Cruz Biotechnology) on Protein G Sepharose 4 Fast Flow beads (GE healthcare). The samples were incubated at 4°C overnight and then washed with progressively more stringent conditions. After the IP, the samples were de-crosslinked and purified using a QIAquick PCR purification kit (Qiagen) and analysed using a qPCR.

### Quantitative PCR

Quantitative PCR was performed with the FastStart Universal SYBR Green Master Mix (Roche) using a 7900HT Fast Real-Time PCR System in a 384-Well Block Module (Applied Biosystems™). Primers used to detect enrichment at the INT sequence and at *ACTA1* gene are listed in Supplementary Material, Table S4. Ct values were analysed using the SDS Software v2.4. The percentage of input reported was obtained by dividing the amount of precipitated DNA for the locus of interest by the amount in the input samples multiplied by 100%.

### Small-pool PCR

Small-pool PCR experiments were performed on DNA isolated from 91-Y, 59B-Y-HDAC5 and 89B-Y-DNMT1 cells grown with ABA or DMSO only for 30 days in the presence of the appropriate selection markers. The SP-PCR protocol used is described in (59). We used primers oVIN-460 and oVIN-1425 (Supplementary Material, Table S4) to amplify the repeat tract, ran the products on a TAE agarose gel and alkaline transferred it on positively charged nylon membrane (MegaProbe). The membranes were then probed with oVIN-100 (5′-CAGCAGCAGCAGCAGCAGCAGCAGCAGCAG) that was end-labelled with ^32^P and exposed to a phosphoscreen and scanned with a Typhoon scanner. For each sample, we first performed serial dilutions to obtain the concentration of amplifiable alleles. We calculated that concentration by using the number of reactions that led to no amplification and used that probability to calculate the average number of alleles per PCR using a Poisson distribution as described (26). We used between 2 and 20 alleles per reaction for the quantifications seen in Table 1 and Supplementary Material, Fig. S4. To quantify repeat instability, membranes were blinded, and a different lab member drew lines at the top and bottom of the most common bands and then counted individual alleles that fell outside of these lines. The number of alleles that contracted or expanded were divided by the total number of alleles amplified as estimated using a Poisson distribution. To look for the changes in the size of the repeat tract, we used a 1 kb DNA ladder to bin the repeats according to their size. Note that we could detect smaller changes in repeat size in the instability analyses shown in Table 1 where we quantified expansions, contractions, and no change, used the most common allele as a reference point. Consequently, in the case where we quantify the change in repeat size (Supplementary Material, Fig. S4), we had lower levels of instability. Moreover, we had fewer alleles analysed as two gels were not photographed prior to transfer onto a membrane and could not be analysed adequately. Unaltered small-pool PCR blots are found in Supplementary Material, Fig. S10. We have noted that cell lines with repeats that are mildly expanded (e.g. 59 CAGs) have fewer contractions than longer ones. This is consistent with studies in the context of DM1 and HD (61), albeit the size threshold for seeing more contractions may be shorter in HEK293-derived cells than in mice.

### Bisulfite sequencing

Bisulfite conversion was done using the EZ DNA Methylation kit from Zymo Research as described before (13). We converted 200 ng of DNA at 50°C for 12 h from each cell line after 30 days of culturing with ABA. We used primer oVIN-2209 and oVIN-2211 to amplify the converted DNA (Supplementary Material, Table S4). The products were then purified using the NucleoSpin PCR Clean-up kit (Macherey-Nagel). We then performed 2 × 250 bp paired-end MiSeq sequencing (Illumina). The sequencing primers are found in Supplementary Material, Table S4. We processed the reads with TrimGalore (github.com/FelixKrueger/TrimGalore) using -q 20 —length 20 –paired. We aligned the reads using QuasR (62) to the GFP transgene sequence. We extracted the methylation levels for each CpG in the amplicon with the qMeth() function in QuasR. We calculated the CpG methylation frequencies by dividing the frequency of methylated CpGs by the total number of CpG and expressed it as a percentage.

### Statistics

We determined statistical significance in the targeting and ChIP experiments using a two-tailed one-way ANOVA. For small-pool PCR we used a χ^2^ test with two degrees of freedom using three categories: expansions, contractions and no change. We used a Fisher’s exact test in the case of the 59B-Y-HDAC5 lines because we found no contractions. Most statistical analyses were done using R studio version 3.4.0, with the exception of the two-way ANOVA, the paired t-tests, and the Mann Whitney U tests, which were done using GraphPad Prims version 8.4.2. We concluded that there was a significant difference when *P* < 0.05.

## Supplementary Material

210830_Yang_et_al_Supplement_ddab255
