# Correction to: Expanded CAG/CTG repeats resist gene silencing mediated by targeted epigenome editing

**DOI:** 10.1093/hmg/ddae167

**Published:** 2024-11-15

*Human Molecular Genetics,* 2022, Vol. 31, No. 3, 1 February 2022, Pages 386–398, https://doi.org/10.1093/hmg/ddab255

In September 2024, the authors contacted the journal to request publication of a corrected Supplementary Fig. 5. The original figure represents data from counting 53 alleles. When the authors recently repeated the experiment counting a higher number of alleles (n = 120) the low levels of expansion in the condition without transcription were not replicated. Instead, the same amount of expansions with and without transcription were observed.

Consequently, the following sentence in the Results under the heading “No evidence that DNMT1 impacts repeat instability by acting in cis,” third paragraph are incorrect: "We saw a transcription- and time-dependent increase in expansions (Supplementary Material, Fig. S5A). Moreover, the addition of 10 μM of RGFP966, a specific HDAC3 inhibitor (27), abolished the transcription-dependent expansions and increased H3ac levels (Supplementary Material, Fig. S5A and B).”

The corrected sentence is: “We saw a time-dependent increase in expansions (Supplementary Material, Fig. S5A), which was suppressed by the addition of 10 μM of RGFP966, a specific HDAC3 inhibitor (27). This treatment also increased H3ac levels (Supplementary Material, Fig. S5B).”

**Supplementary Figure 5**


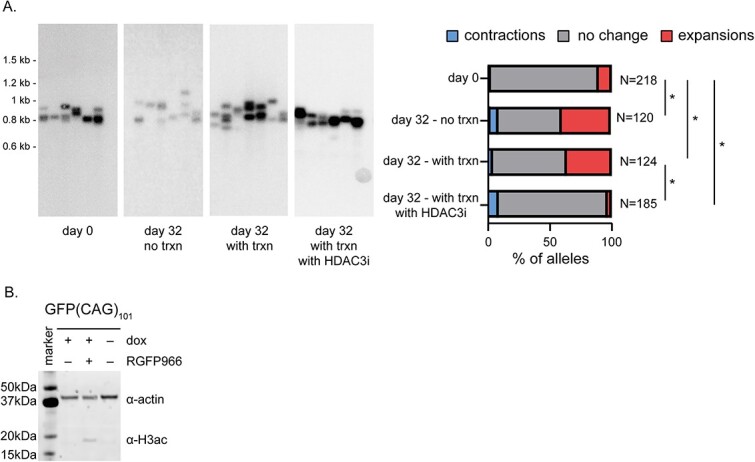


These corrected results do not change the main conclusions of the article.

