# Supplementary material for: Expanded CAG/CTG repeats resist gene silencing mediated by targeted epigenome editing
Source: Hum Mol Genet. 2021 Sep 7;31(3):386–98. doi: 10.1093/hmg/ddab255 (PMC8825355; doi:10.1093/hmg/ddab255)
Supplement: 210830_Yang_et_al_Supplement_ddab255 [file 210830_yang_et_al_supplement_ddab255.docx]

Supplementary Material

Expanded CAG/CTG Repeats Resist Gene Silencing

Mediated by Targeted Epigenome Editing

Bin Yang^1^, Alicia C. Borgeaud^1,2^, Marcela Buřičová^3^, Lorène Aeschbach^1^, Oscar Rodríguez-Lima^1^, Gustavo A. Ruiz Buendía^1^, Cinzia Cinesi^1^, Alysha S. Taylor^3^, Tuncay Baubec^4^, and Vincent Dion^3^*

1: Center for Integrative Genomics, Faculty of Biology and Medicine, University of Lausanne, 1015 Lausanne, Switzerland.

2: Current address: MRC Laboratory of Molecular Biology, Francis Crick Avenue, Cambridge Biomedical Campus, Cambridge CB2 0QH, United Kingdom.

3: UK Dementia Research Institute at Cardiff University, Hadyn Ellis Building, Maindy Road, CF24 4HQ, Cardiff, United Kingdom.

5: Department of Molecular Mechanisms of Disease, University of Zurich, 8057 Zurich, Switzerland.

*: Corresponding author: [dionv@cardiff.ac.uk](mailto:dionv@cardiff.ac.uk)

**Table S1: Cell lines**

| Cell line name | Parent cell line | Transgenes | Plasmid used | Integration method | Resistance marker | Reference |
| --- | --- | --- | --- | --- | --- | --- |
| T-REX Flp-in | HEK293 | pFRT/*lac*Zeo | - | - | Blasticidin  Zeocin | Thermo Fisher |
|  |  | Tetracycline Repressor | pcDNA6/TR | - |  |  |
| GFP(CAG)_0_ | T-REX Flp-in | GFP(CAG)_0_ | pGFP(CAG)_0_ | Flp-mediated integration | Blasticidin  Hygromycin | (1) |
| GFP(CAG)_0_B | GFP(CAG)_0_ | GFP(CAG)_0_ | pGFP(CAG)_0_ | Flp-mediated integration | Blasticidin  Puromycin  Hygromycin | This study |
|  |  | ParB-ABI* | pBY-008 | Random integration |  |  |
| GFP-INT | T-REX Flp-in | GFP-INT-CAG_0_ | pVIN-221 | Flp-mediated integration | Blasticidin  Hygromycin | This study |
| GFP-INT-B | GFP-INT | GFP-INT-CAG_0_ | pVIN-221 | Flp-mediated integration | Blasticidin  Puromycin  Hygromycin | This study |
|  |  | ParB-ABI* | pBY-008 | Random integration |  |  |
| GFP-INT-40 | T-REX Flp-in | GFP-INT-CAG_40_ | pVIN-117 | Flp-mediated integration | Blasticidin  Hygromycin | This study |
| 40-B | GFP-INT-40 | GFP-INT-CAG_40_ | pVIN-117 | Flp-mediated integration | Blasticidin  Puromycin  Hygromycin | This study |
|  |  | ParB-ABI* | pBY-008 | Random integration |  |  |
| HEKB | T-REX Flp-in | ParB-ABI* | pBY-008 | Random integration | Blasticidin  Zeocin  Puromycin | This study |
| HEKB-Y | HEKB | ParB-ABI* | pBY-008 | Random integration | Blasticidin  Zeocin  Puromycin  Neomycin | This study |
|  |  | PYL^†^ | pAB-NEO-PYL |  |  |  |
| 16B-Y | HEKB-Y | ParB-ABI* | pBY-008 | Random integration | Blasticidin  Puromycin  Neomycin  Hygromycin | This study |
|  |  | PYL^†^ | pAB-NEO-PYL |  |  |  |
|  |  | GFP-INT-CAG_16_ | pBY-050 | Flp-mediated integration |  |  |
| 91B-Y | HEKB-Y | ParB-ABI* | pBY-008 | Random integration | Blasticidin  Puromycin  Neomycin  Hygromycin | This study |
|  |  | PYL^†^ | pAB-NEO-PYL |  |  |  |
|  |  | GFP-INT-CAG_89_ | pBY-018 | Flp-mediated integration |  |  |

*: Contains 3xHA tag and a NLS. ^†^: Contains 3xFLAG and a NLS.

**Table S1 (continued): Cell lines**

| Cell line name | Parent cell line | Transgenes | Plasmid used | Integration method | Resistance marker | Reference |
| --- | --- | --- | --- | --- | --- | --- |
| HEKB-Y-HDAC3 | HEKB | ParB-ABI* | pBY-008 | Random integration | Blasticidin  Zeocin  Puromycin  Neomycin | This study |
|  |  | PYL-HDAC3^†^ | pAB(EXPR-PYL-HDAC3-NEO) |  |  |  |
| 16B-Y-HDAC3 | HEKB-Y-HDAC3 | ParB-ABI* | pBY-008 | Random integration | Blasticidin  Puromycin  Neomycin  Hygromycin | This study |
|  |  | PYL-HDAC3^†^ | pAB(EXPR-PYL-HDAC3-NEO) |  |  |  |
|  |  | GFP-INT-CAG_16_ | pBY-050 | Flp-mediated integration |  |  |
| 89B-Y-HDAC3 | HEKB-Y-HDAC3 | ParB-ABI* | pBY-008 | Random integration | Blasticidin  Puromycin  Neomycin  Hygromycin | This study |
|  |  | PYL-HDAC3^†^ | pAB(EXPR-PYL-HDAC3-NEO) |  |  |  |
|  |  | GFP-INT-CAG_89_ | pBY-018 | Flp-mediated integration |  |  |
| HEKB-Y-HDAC5 | HEKB | ParB-ABI* | pBY-008 | Random integration | Blasticidin  Zeocin  Puromycin  Neomycin | This study |
|  |  | PYL-HDAC5^†^ | pAB(EXPR-PYL-HDAC5-NEO) |  |  |  |
| 16B-Y-HDAC5 | HEKB-Y-HDAC5 | ParB-ABI* | pBY-008 | Random integration | Blasticidin  Puromycin  Neomycin  Hygromycin | This study |
|  |  | PYL-HDAC5^†^ | pAB(EXPR-PYL-HDAC5-NEO) |  |  |  |
|  |  | GFP-INT-CAG_16_ | pBY-050 | Flp-mediated integration |  |  |
| 59B-Y-HDAC5 | HEKB-Y-HDAC5 | ParB-ABI* | pBY-008 | Random integration | Blasticidin  Puromycin  Neomycin  Hygromycin | This study |
|  |  | PYL-HDAC5^†^ | pAB(EXPR-PYL-HDAC5-NEO) |  |  |  |
|  |  | GFP-INT-CAG_59_ | pBY-018 | Flp-mediated integration |  |  |

*: Contains 3xHA tag and a NLS. ^†^: Contains 3xFLAG and a NLS.

**Table S1 (continued): Cell lines**

| HEKB-Y-DNMT1 | HEKB | ParB-ABI* | pBY-008 | Random integration | Blasticidin  Zeocin  Puromycin  Neomycin | This study |
| --- | --- | --- | --- | --- | --- | --- |
|  |  | PYL-DNMT1^†^ | pAB(EXPR-PYL-DNMT1-NEO) |  |  |  |
| 16B-Y-DNMT1 | HEKB-Y-DNMT1 | ParB-ABI* | pBY-008 | Random integration | Blasticidin  Puromycin  Neomycin  Hygromycin | This study |
|  |  | PYL-DNMT1^†^ | pAB(EXPR-PYL-DNMT1-NEO) |  |  |  |
|  |  | GFP-INT-CAG_16_ | pBY-050 | Flp-mediated integration |  |  |
| 89B-Y-DNMT1 | HEKB-Y-DNMT1 | ParB-ABI* | pBY-008 | Random integration | Blasticidin  Puromycin  Neomycin  Hygromycin | This study |
|  |  | PYL-DNMT1^†^ | pAB(EXPR-PYL-DNMT1-NEO) |  |  |  |
|  |  | GFP-INT-CAG_89_ | pBY-018 | Flp-mediated integration |  |  |

*: Contains 3xHA tag and a NLS. ^†^: Contains 3xFLAG and a NLS.

**Table S2: Plasmids used for transient transfection experiments**

| Name | Description | Source |
| --- | --- | --- |
| pBY-008 | Expresses ParB-ABI with 3xHA and a SV40 NLS | This study |
| pBY-022 | Expresses PYL fused to 3xFLAG and a SV40 NLS. Also serves as a destination vector for making fusions | This study |
| pBY-006 | Expresses PYL-HDAC3 with 3xFLAG and a SV40 NLS | This study |
| pBY-017 | Expresses PYL-HDAC5 with 3xFLAG and a SV40 NLS | This study |
| pAB-HDAC5(mut-H-A) | Expresses PYL-HDAC5 H1006A with 3xFLAG and a SV40 NLS | This study |
| pAB-HDAC5(mut-H-Y) | Expresses PYL-HDAC5 H1006Y with 3xFLAG and a SV40 NLS | This study |
| pAB(EXPR-HDAC5-trunc1) | Expresses PYL-HDAC5 aa 1-275 with 3xFLAG and a SV40 NLS | This study |
| pAB-EXPR(PYL-cat_dom_HDAC5) | Expresses the PYL-HDAC5 catalytic domain with 3xFLAG and a SV40 NLS | This study |
| pAB-EXPR(PYL-Nterm_dom_HDAC5) | Expresses the PYL-HDAC5 N terminal domain fused to 3xFLAG and a SV40 NLS | This study |
| pAB(EXPR-PYL-DNMT1-NEO) | Expresses PYL-DNMT1 with 3xFLAG and a SV40 NLS | This study |

**Table S3: Antibodies**

| Epitope | Company | Catalog number | Dilution | Assay |
| --- | --- | --- | --- | --- |
| FLAG | Sigma-Aldrich | F1804-5MG | 3 μg per IP | ChIP |
|  |  |  | 1:1000 | WB |
| HA | Sigma-Aldrich | 12158167001 | 1:2000 | WB |
| IgG | Santa Cruz Biotechnology | sc-69786 | 3 μg per IP | ChIP |
| Pan-acetylation of H3 | Merck | #06-599 | 3 μg per IP | ChIP |
| Histone H3 | Abcam | ab1791 | 3 μg per IP | ChIP |
| Actin | Sigma-Aldrich | A2066-.2ML | 1:2000 | WB |

**Table S4: Primers using in this study**

| Oligo | Sequence* | Targeted locus | Reference |
| --- | --- | --- | --- |
| oVIN-0459 | 5’ AAGAGCTTCCCTTTACACAACG | GFP transgene | (2) |
| oVIN-0460 | 5’ TCTGCAAATTCAGTGATGC | GFP transgene | (2) |
| oVIN-1425 | 5’ GACCTCATACGAAGATAGGCTT | GFP transgene | This study |
| oVIN-0969 | 5’ TGAATACCATGCGCTCTA | *INT* | This study |
| oVIN-0970 | 5’ GCCGTTCGTGGCAGAGAT | *INT* | This study |
| oVIN-1075 | 5’ AGCGCGGCTACAGCTTCAC | *ACTA1* | This study |
| oVIN-1076 | 5’ CAGCCGTGGCCATCTCTT | *ACTA1* | This study |
| oVIN-2209 | 5’TCGTCGGCAGCGTCAGATGTGTATAAGAGACAG TTTGTATYGAGGGGTTTGATGGGGGG | *INT* bisulfite | This study |
| oVIN-2211 | 5’GTCTCGTGGGCTCGGAGATGTGTATAAGAGACAG ACTTTATACRCATAAACAACCACTCTTC | *INT* bisulfite | This study |

*** :** underlined: sequencing adapters. Y: pyrimidines, R: purines.

**
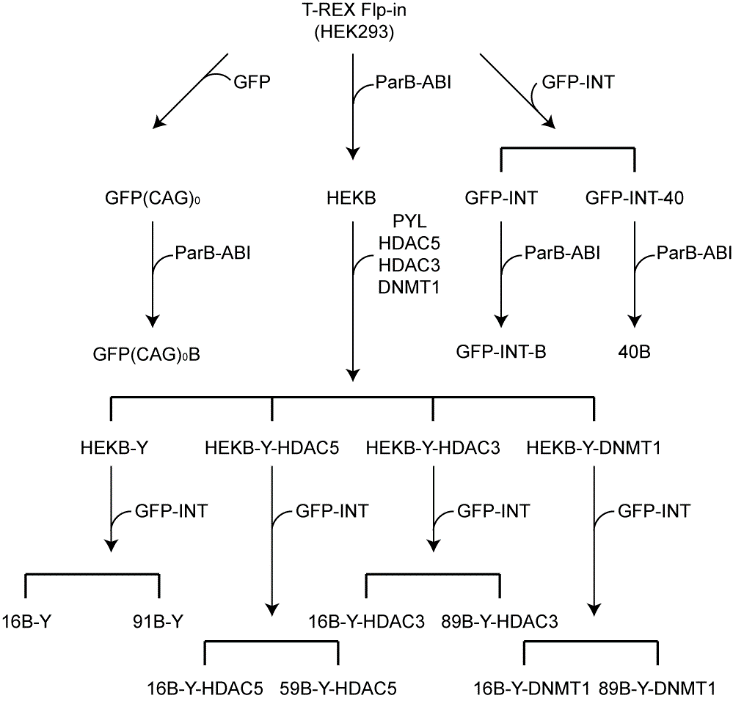
**

**Fig. S1. Parentage of the cell lines described in this study.** All cell lines are clonal. Details of each plasmid integrated, the methods of doing so, and the details of the cell lines are found in Tables S1.

**
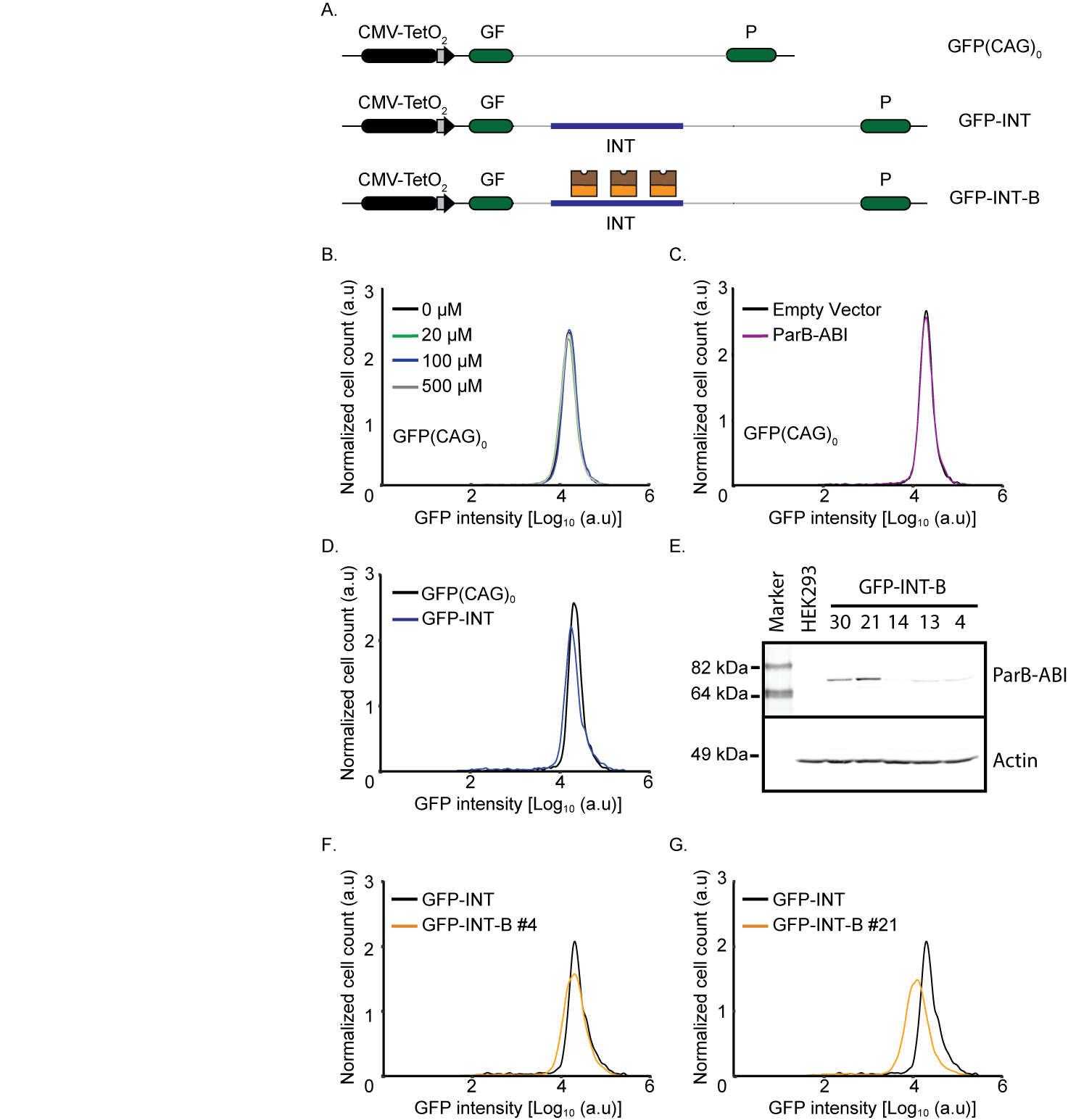
Fig. S2.** **Effect of components of PInT on GFP expression.** A) Cartoon of cell lines used. B) Representative flow cytometry profiles of GFP(CAG)_0_ cells treated with increasing concentrations of ABA dissolved into the same volume of DMSO. C) Representative flow cytometry profiles of GFP(CAG)_0_ cell lines transfected with a plasmid expressing ParB-ABI or an empty vector. D) Comparison of GFP expression between GFP(CAG)_0_ and GFP-INT cells. E) Western blots (against HA) of GFP-INT-B clones showing varying amounts of ParB-ABI in the different clones. F) Flow cytometry profiles of GFP-INT-B clone #4, which expresses low levels of ParB-ABI. G) Representative flow cytometry profile of GFP-INT-B clone #21 expressing a larger amount of ParB-ABI. Note that the GFP-INT parent profile is the same in panels F and G because the GFP expression of both clones was done on the same day using the same parent cell line as control.

**
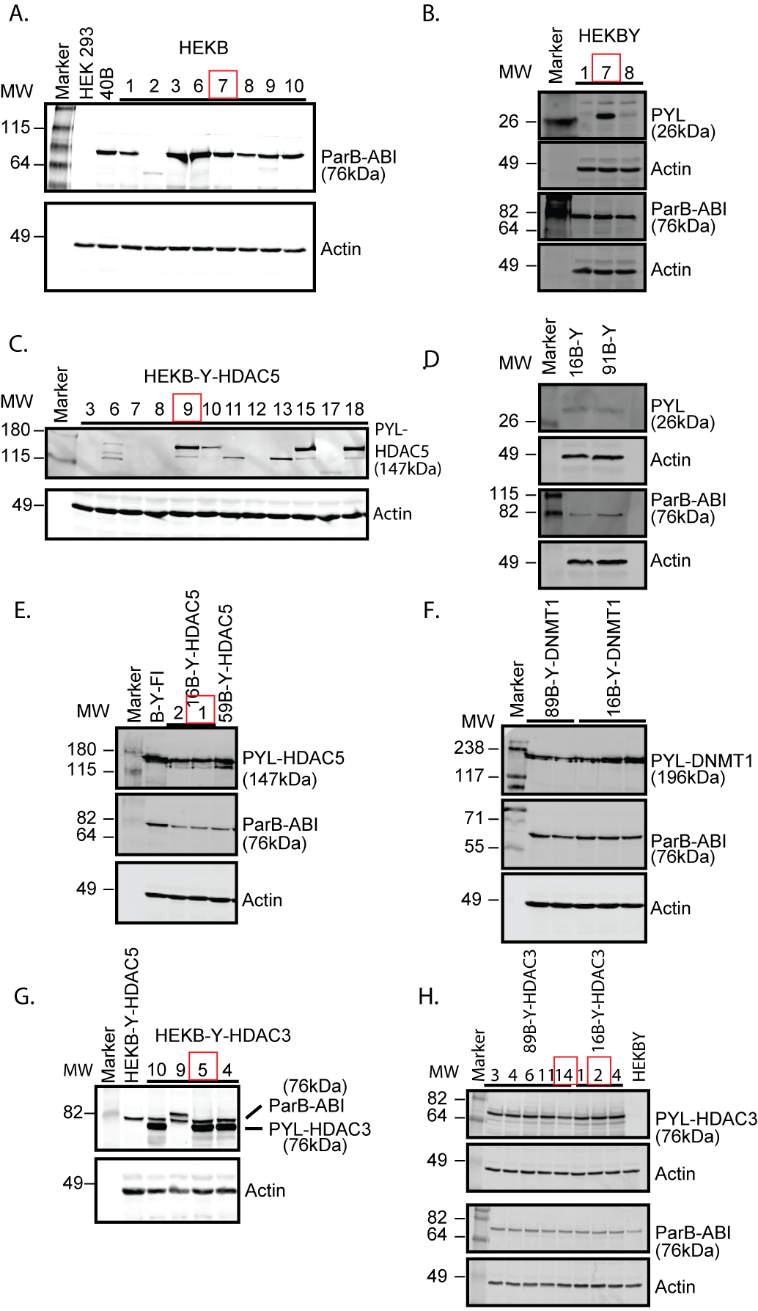
Fig. S3. Cell line making and characterization.** The levels of ParB-ABI and PYL-fusions by western blotting using antibodies against HA and FLAG, respectively. The red boxes identify the clones that were used subsequently. A) ParB-ABI levels in HEKB clones. B) PYL and ParB-ABI levels in HEKBY cells. C) PYL-HDAC5 in HEKB-Y-HDAC5 cells. D) PYL and ParB-ABI levels in 16B-Y and 91B-Y cells E) PYL-HDAC5 and ParB-ABI levels in 16B-Y-HDAC5 and 59B-Y-HDAC5 cells. F) PYL-DNMT1 and ParB-ABI levels in 89B-Y-DNMT1 and 16B-Y-DNMT1 cells. G) PYL-HDAC3 and ParB-ABI levels in HEKBYH3 cells. H) PYL-HDAC3 and ParB-ABI levels in 16B-Y-HDAC3 and 89B-Y-HDAC3 cells. The MW marker used was the BenchMark™ Pre-stained Protein Standard, except in (F), where the HiMark™ Pre-stained Protein Standard was used.


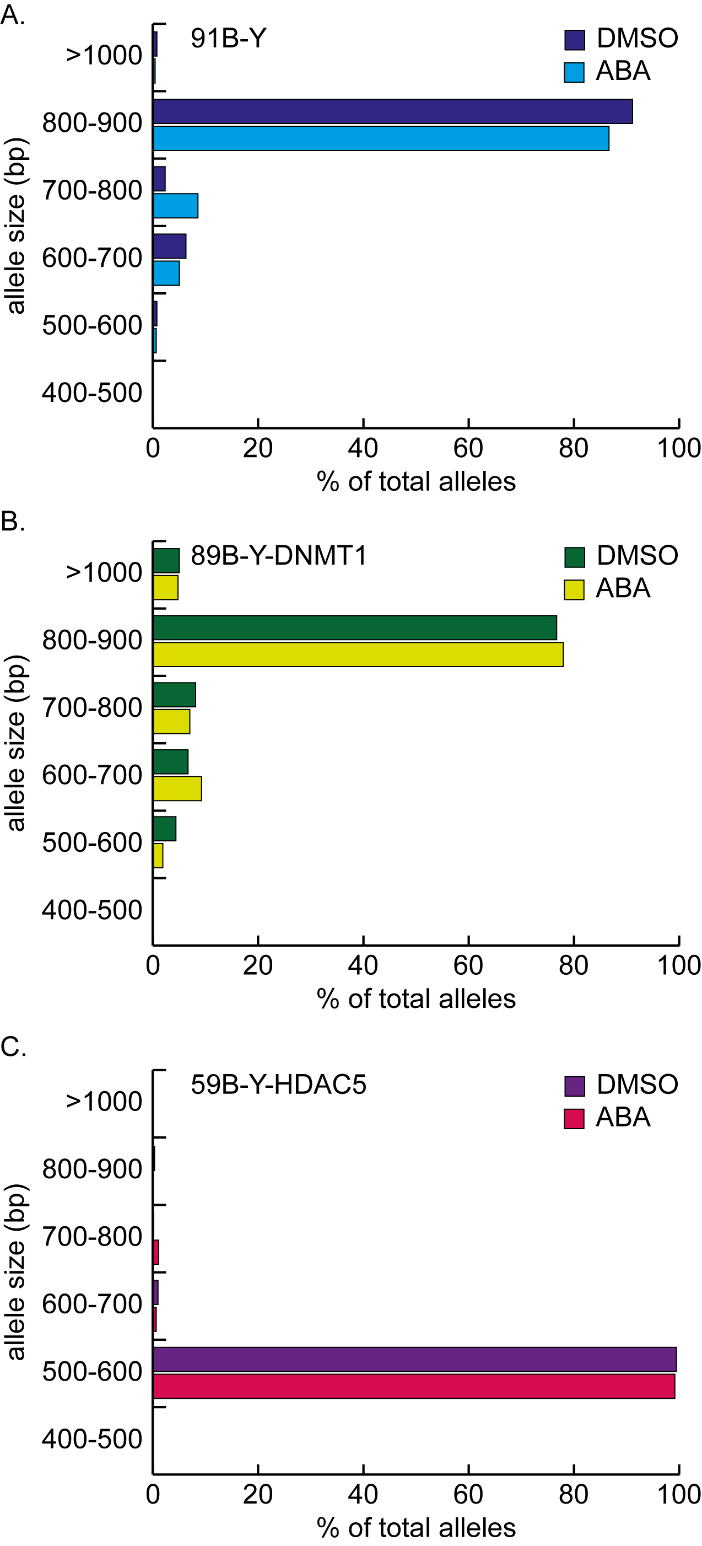
**Fig. S4: Quantification of the allele size changes upon targeting.** We re-analysed the data presented in Table 1 and binned the alleles based on their size using a 1kb ladder and performed Mann-Whitney u tests to look for differences between ABA treatment and the vehicle alone. A) 91B-Y cells. P=0.021. B) 89-B-Y-DNMT1. P=0.77. C) 59B-Y-HDAC5. P=0.70.

**
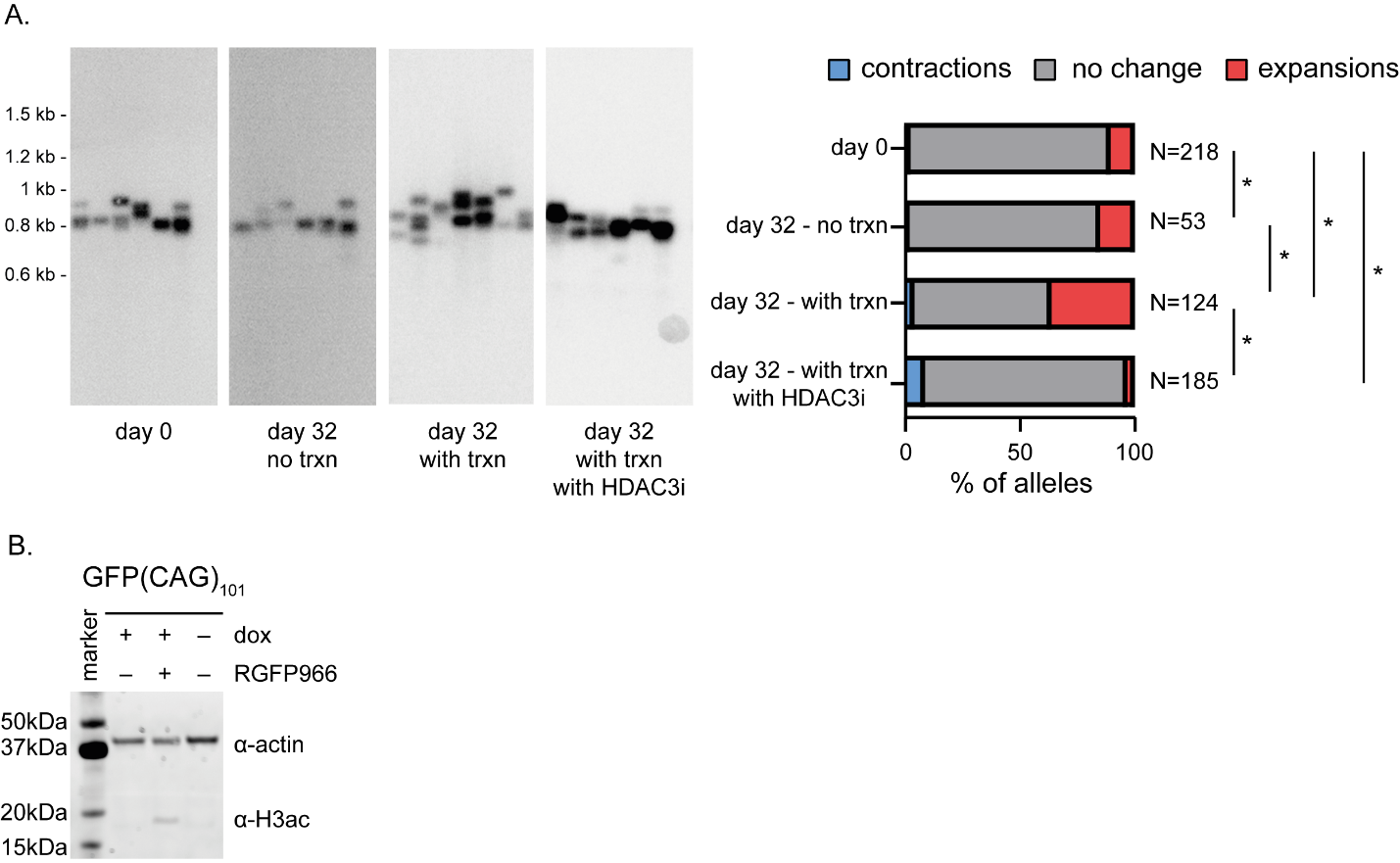
**

**Fig. S5: Time-dependent, transcription-dependent, and HDAC3-dependent effect on repeat instability in GFP(CAG)_101_ cells.** A) Representative small-pool PCR membranes (left) for a starting population (day 0), 32 days later in the presence of DMSO (without transcription (trxn)), with doxycycline (with transcription (trxn)), and in the presence of both dox and 10 µM RGFP966 (HDAC3i). The quantification is seen on the right. *: P ≤ 0.003, using a χ^2^ tests. B) Western blot showing an increase in acetylated histone H3 in GFP(CAG)_101_ cells treated 10 µM RGFP966 compared to DMSO alone.

**
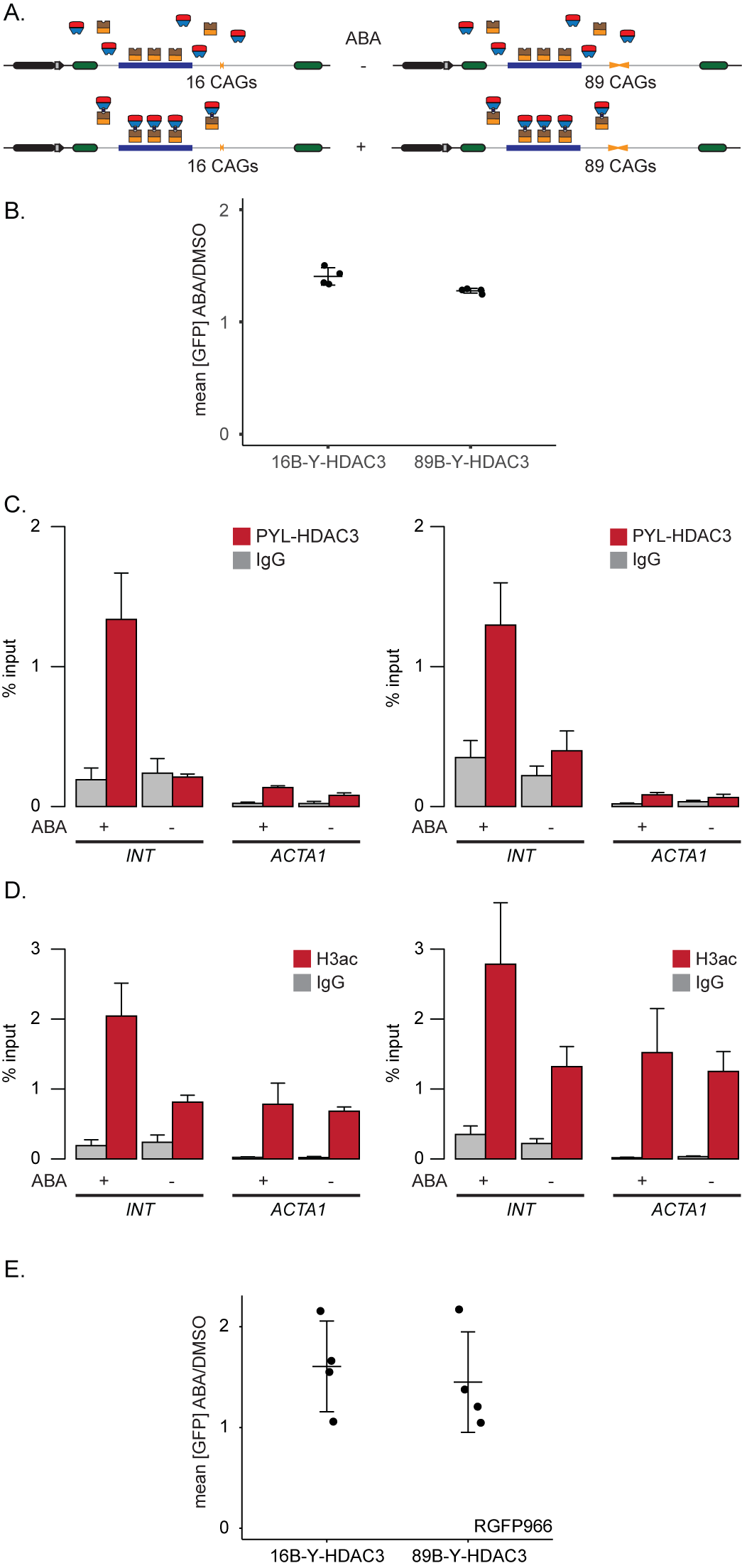
Fig. S6: Targeting of PYL-HDAC3 increases GFP expression independently of its catalytic activity.** A) Schematic representation of the nB-Y-HDAC3 cells. B) Quantification of GFP expression in nB-Y-HDAC3 cells with ABA or DMSO (16B-Y-HDAC3: N=4; 89B-Y-HDAC3: N=4). The error bars are the standard error around the indicated mean. C) ChIP-qPCR experiments using an antibody against PYL-HDAC3 fusion (FLAG) at the INT and *ACTA1* loci in the presence of ABA or DMSO. Left, 16B-Y-HDAC3 cells (N=4), Right, 89B-Y-HDAC3 cells (N=4). The error bars are the standard error. D) ChIP-qPCR experiments using an antibody against pan-acetylated H3 (H3ac) at the INT and *ACTA1* loci in the presence of ABA or DMSO. Left, 16B-Y-HDAC3 cells (N=4), Right, 89B-Y-HDAC3 cells (N=4). The error bars are the standard error. E) Quantification of GFP expression in nBYH3 cells with ABA or DMSO and treated with RGFP966 (16B-Y-HDAC3: N=4; 89B-Y-HDAC3: N=4). The error bars are the standard error around the indicated mean.

**
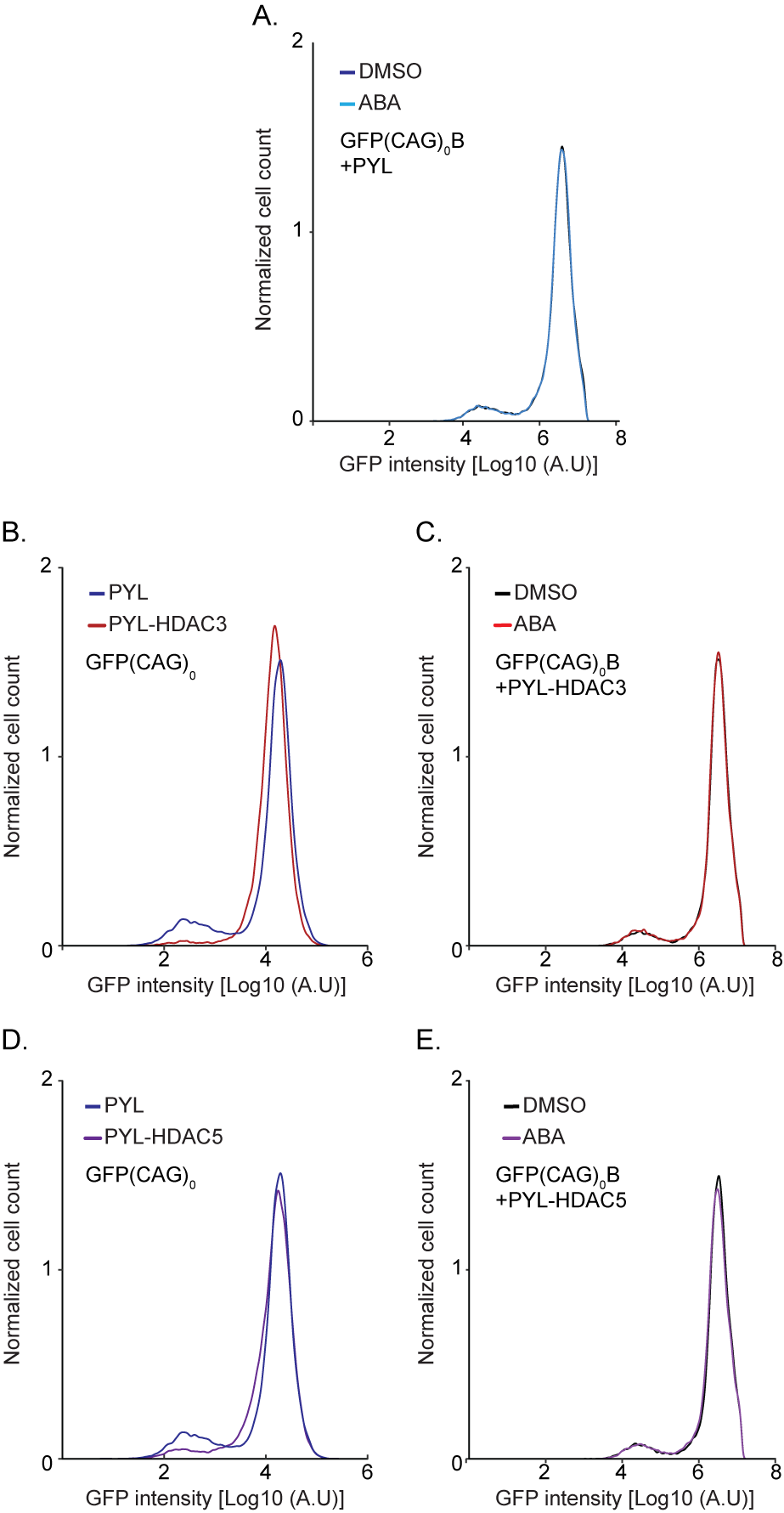
Fig. S7. The functionality of PYL fusions in GFP-INT-B cells and GFP(CAG)_0_B cells.** A) Representative flow cytometry profiles after transient overexpression of PYL in GFP(CAG)_0_B cells with and without ABA. B) Representative flow cytometry data overexpressing PYL or PYL-HDAC3 in GFP(CAG)_0_ cells. Similar results were obtained using GFP-INT-B cells. C) Representative flow cytometry profiles of GFP(CAG)_0_B cells transiently transfected with PYL-HDAC3 with or without ABA. D) Representative flow cytometry data overexpressing PYL or PYL-HDAC5 in GFP(CAG)_0_ cells. (E) Representative flow cytometry profiles of GFP(CAG)_0_B cells transiently transfected with PYL-HDAC5 with or without ABA.

**
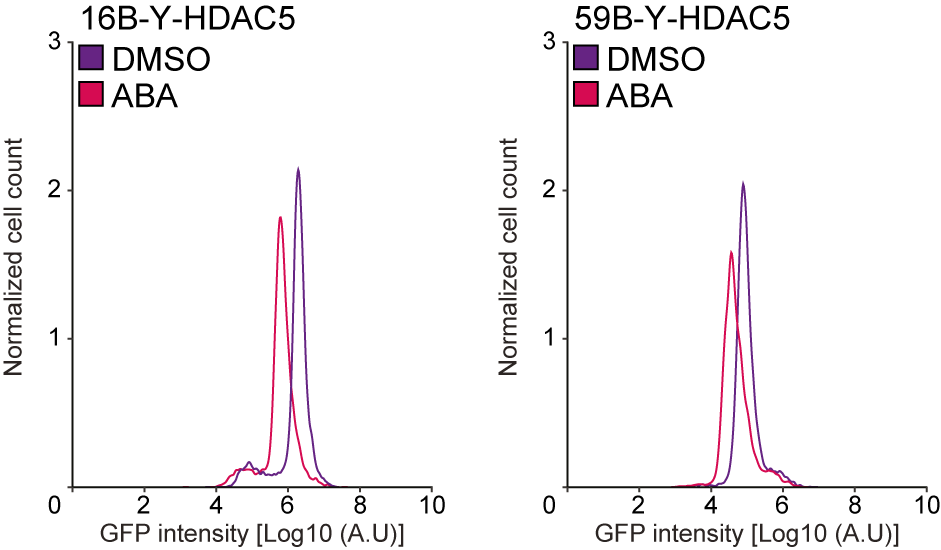
Fig. S8: Representative examples of the effect of PYL-HDAC5 on GFP intensity.** Representative flow cytometry data showing a shift in GFP expression upon addition of ABA to the culture medium in 16B-Y-HDAC5 versus 59B-Y-HDAC5. Note that the shift in expression is greater in 16B-Y-HDAC5 than in 59B-Y-HDAC5.**
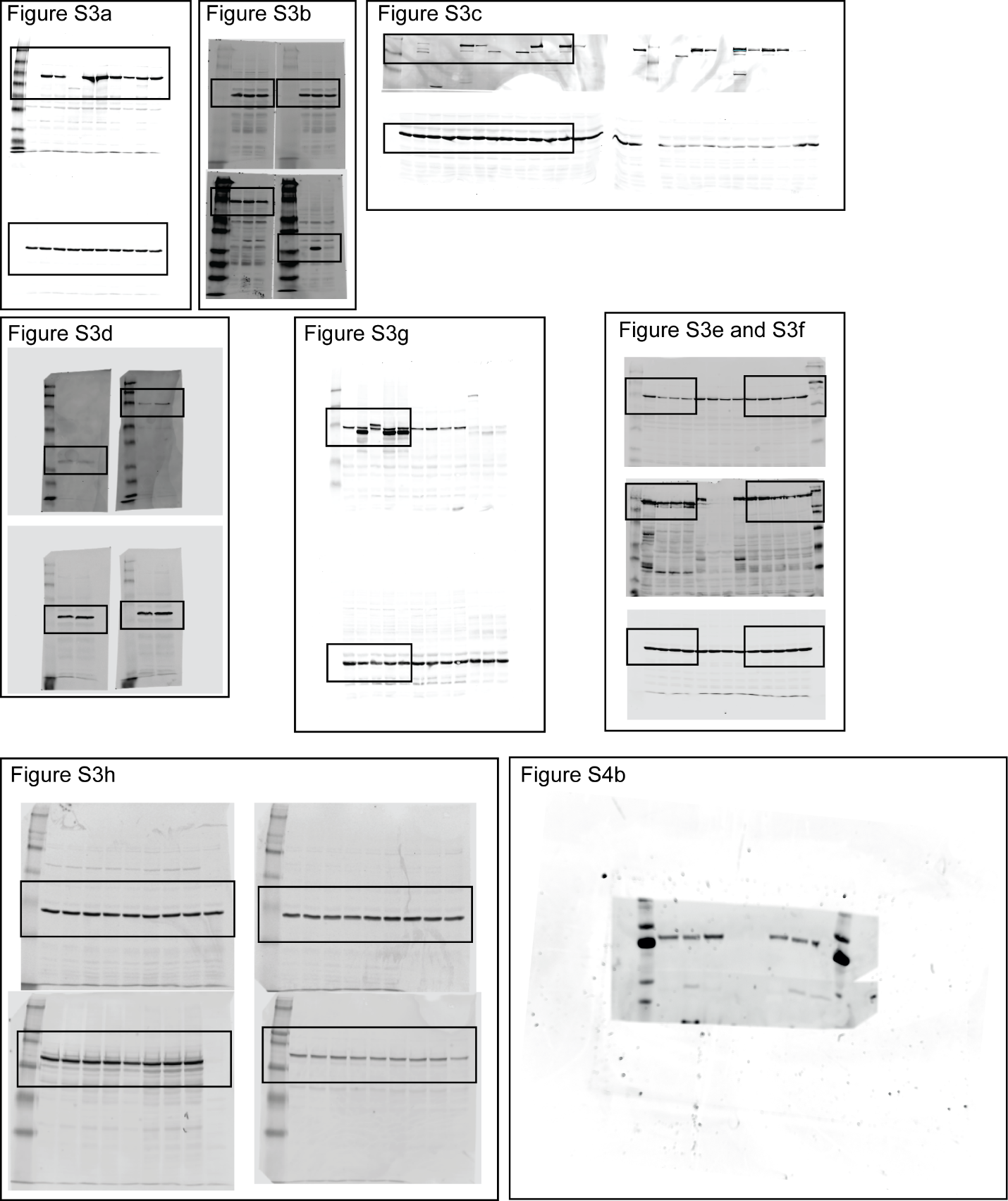
**

**Fig. S9:** **Unaltered full western blots.** Uncropped Western blot membranes from figures S2, S3 and S4b. Black boxes indicate where the blots were cropped.


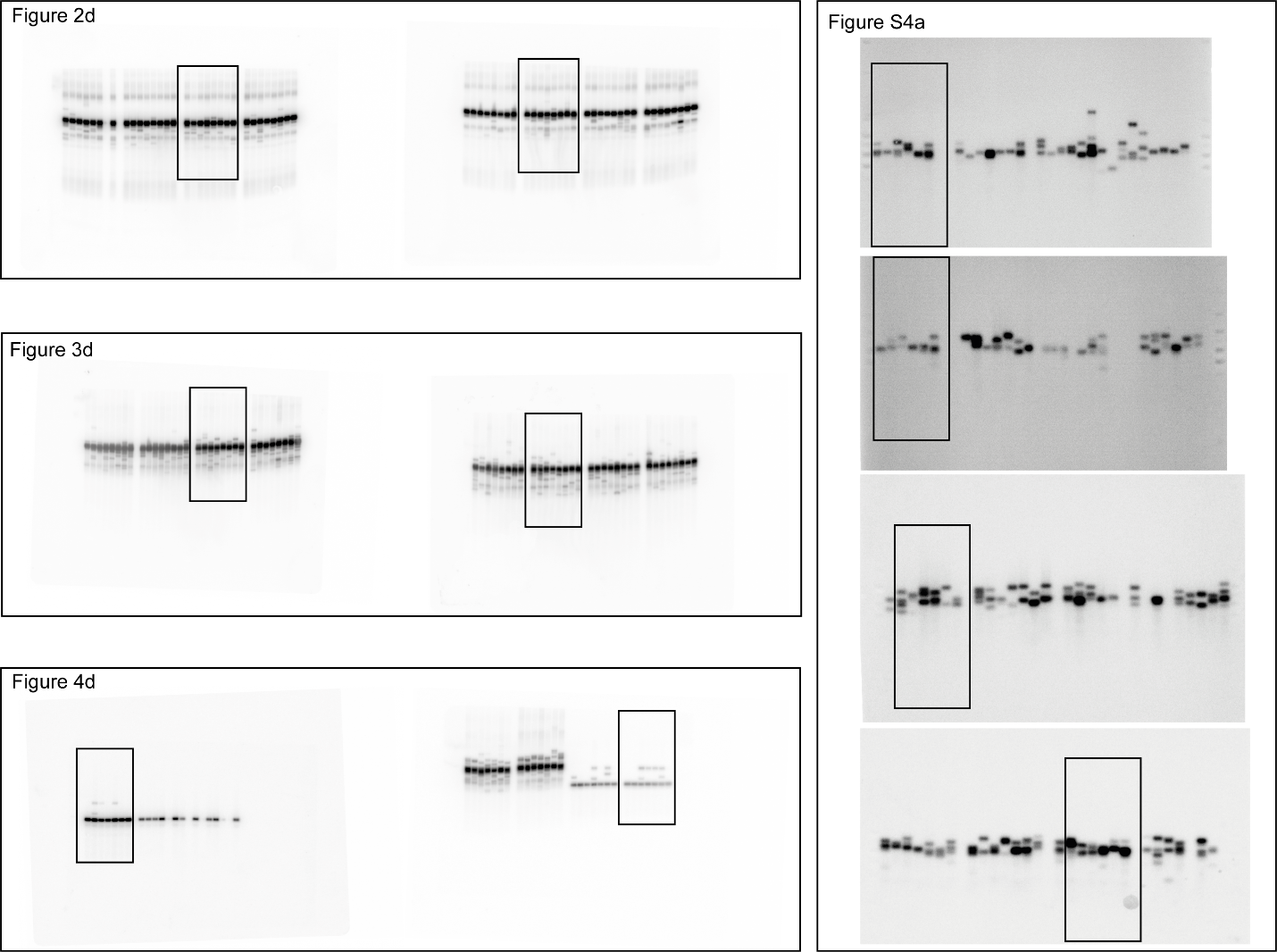
**Fig. S10:** **Uncropped small-pool PCR blots.** Shown are the uncropped SP-PCR membranes displayed in Figures 2d, 3d, 4d, and S4a. Black boxes indicate where the blots were cropped.

**Supplementary references:**

1. Santillan, B. A., Moye, C., Mittelman, D. and Wilson, J. H. (2014) GFP-based fluorescence assay for CAG repeat instability in cultured human cells. *PLoS One*, **9**, e113952.

2. Cinesi, C., Aeschbach, L., Yang, B. and Dion, V. (2016) Contracting CAG/CTG repeats using the CRISPR-Cas9 nickase. *Nat. Commun.*, **7**, 13272.
